# Histone isoform H2A1H promotes attainment of distinct physiological states by altering chromatin dynamics

**DOI:** 10.1186/s13072-017-0155-z

**Published:** 2017-10-18

**Authors:** Saikat Bhattacharya, Divya Reddy, Vinod Jani, Nikhil Gadewal, Sanket Shah, Raja Reddy, Kakoli Bose, Uddhavesh Sonavane, Rajendra Joshi, Sanjay Gupta

**Affiliations:** 1 Epigenetics and Chromatin Biology Group, Gupta Lab, Cancer Research Institute, Advanced Centre for Treatment, Research and Education in Cancer (ACTREC), Tata Memorial Centre, Kharghar, Navi Mumbai, MH 410210 India; 2 Integrated Biophysics and Structural Biology Lab, Cancer Research Institute, Advanced Centre for Treatment, Research and Education in Cancer (ACTREC), Tata Memorial Centre, Kharghar, Navi Mumbai, MH 410210 India; 3 BTIS, Cancer Research Institute, Advanced Centre for Treatment, Research and Education in Cancer (ACTREC), Tata Memorial Centre, Kharghar, Navi Mumbai, MH 410210 India; 4 Homi Bhabha National Institute, Training School Complex, Anushakti Nagar, Mumbai, MH 400085 India; 5 Bioinformatics Group, Centre for Development of Advanced Computing (C-DAC), University of Pune Campus, Pune, MH 411007 India; 6 Stowers Institute for Medical Research, Kansas City, MO 64110 USA

**Keywords:** Cancer, Chromatin, Differentiation, Histone, Nucleosome

## Abstract

**Background:**

The distinct functional effects of the replication-dependent histone H2A isoforms have been demonstrated; however, the mechanistic basis of the non-redundancy remains unclear. Here, we have investigated the specific functional contribution of the histone H2A isoform H2A1H, which differs from another isoform H2A2A3 in the identity of only three amino acids.

**Results:**

H2A1H exhibits varied expression levels in different normal tissues and human cancer cell lines (H2A1C in humans). It also promotes cell proliferation in a context-dependent manner when exogenously overexpressed. To uncover the molecular basis of the non-redundancy, equilibrium unfolding of recombinant H2A1H-H2B dimer was performed. We found that the M51L alteration at the H2A–H2B dimer interface decreases the temperature of melting of H2A1H-H2B by ~ 3 °C as compared to the H2A2A3-H2B dimer. This difference in the dimer stability is also reflected in the chromatin dynamics as H2A1H-containing nucleosomes are more stable owing to M51L and K99R substitutions. Molecular dynamic simulations suggest that these substitutions increase the number of hydrogen bonds and hydrophobic interactions of H2A1H, enabling it to form more stable nucleosomes.

**Conclusion:**

We show that the M51L and K99R substitutions, besides altering the stability of histone–histone and histone–DNA complexes, have the most prominent effect on cell proliferation, suggesting that the nucleosome stability is intimately linked with the physiological effects observed. Our work provides insights into the molecular basis of the non-redundancy of the histone H2A isoforms that are being increasingly reported to be functionally important in varied physiological contexts.

**Electronic supplementary material:**

The online version of this article (doi:10.1186/s13072-017-0155-z) contains supplementary material, which is available to authorized users.

## Background

Histones are a class of highly conserved basic proteins that package the genome. The core histones are comprised of H2A, H2B, H3 and H4 which form the octameric protein core of the fundamental repeating unit of chromatin, the nucleosome. Around this core, ~147 bp of DNA is wrapped to form the nucleosome core particle (NCP) [1]. Further compaction of the chromatin is achieved with the aid of the linker histone H1 [2].

The canonical histone proteins are synthesized during the S-phase, and to meet up with their high demand during DNA replication, genes that encode them are present in clusters. There are three clusters of canonical histone genes present in humans at chromosome numbers 1 and 6. Notably, differences in the primary sequence are observed amongst the histone proteins encoded by these genes. For the sake of clarity, these are termed as the histone isoforms in this manuscript. In humans, there are 17 genes for H2A that code for 12 isoforms [3, 4]. Likewise, there are 13 genes for H2A in rats that code for 9 isoforms (most are “predicted”).

The histone isoform genes are named based on their identity and location in the genome. In the name of the gene, the first part refers to the cluster (HIST1—cluster 1, HIST2—cluster 2, HIST3—cluster 3), the second part of the gene name introduces the type of histone (H2A, H2B, H3, H4, H1), and the third part indicates the alphabetical order within each cluster (centromere distal to proximal). Therefore, HIST1H2AB refers to the second histone H2A gene in the histone cluster 1 and HIST2H2AB refers to the second histone H2A gene in the histone cluster 2. The proteins coded by these genes, however, were not referred to as systematically. Traditionally, the histone H2A isoforms were broadly classified into two categories, H2A.1 and H2A.2, based on the difference in their mobility on AUT (acetic acid, urea, Triton X-100)–PAGE gels. The H2A isoforms that migrated slowly were collectively termed H2A.1 and the isoforms that migrated faster were collectively referred to as H2A.2 [5]. The difference in migration arises due to the L51M alteration in H2A. Leucine binds more Triton X, and hence, the H2A isoforms with L51 migrate slower than isoforms with M51 residue. However, as each of these two bands may be constituted of multiple proteins, this system of referring to isoforms can be misleading. Especially considering the growing evidence of the changes in the expression level of the isoforms, a better way to name them would be to maintain consistency with their gene nomenclature. For example, the protein coded by HIST1H2AB will be referred to as H2A1B. If two genes code for the same protein as in the case of HIST1H2AB and HIST1H2AE, the protein will be referred to as H2A1B/E. Hence, in rats, the proteins H2A3, H2A4, H2A1F, H2A1K, H2A1H and H2A1C (H2AE-like, H2A1I, H2A1N) constitute the H2A.1 isoforms, and the H2A2B, H2A2C and H2A2A3 proteins belong to H2A.2 isoforms (see Additional file 1: Figure S1 for the alignment).

The histone isoforms were considered functionally redundant for a long time considering the similarity in their amino acid sequences. Interestingly, though, the H2A isoforms have been reported to be differentially expressed in a variety of physiological states. For instance, the proportion of the H2A.1 and H2A.2 isoforms in rats has been shown to decrease during the course of development, differentiation and aging [6–8]. An earlier report from our laboratory revealed the overexpression of the H2A.1 isoforms during the sequential stages of rat hepatocellular carcinoma [9]. The expression level of the isoform H2A1C in humans has been reported to alter in pathological states. Expression of the H2A1C isoform was reported to be downregulated in chronic lymphocytic leukemia (CLL) and gall bladder cancer cells [10, 11]. Interestingly, later on in a larger cohort of samples, H2A1C expression was conversely reported to be upregulated in CLL [12]. Also, H2A1C was found to be upregulated in non-small cell lung carcinoma [13]. The levels of H2A1C, in particular, have been reported to change in other diseases including human papillomaviruses hyperplasia, AIDS and multiple sclerosis [14, 15]. Collectively, these reports demonstrate the altered expression of the H2A isoforms in different pathophysiological states. The question now is whether the observed changes are merely a consequence of the change in the state or these isoforms also contribute to the attainment of such states. One report that aims to address this question showed that specific knockdown of H2A1C leads to a marked increase in cell proliferation. This effect is not observed on depleting the other abundant isoforms like H2A1B/E [10]. However, how the histone isoforms impart their non-redundant effects remains unclear.

Here, we show that the expression level of the H2A1H/H2A1C isoform markedly varies in different tissues in addition to being generally upregulated in many cancer cell lines. We provide further evidence that H2A1H (encoded by HIST1H2AH, accession number: NM_001315492.1) provides a growth advantage to cells; however, this effect is context dependent. Importantly, with the help of in vitro and in silico studies, we demonstrate that H2A1H forms more stable nucleosomes than the H2A.2 isoform H2A2A3 (encoded by HIST2H2AA3, accession number: NM_001315493.1), and this is speculated to confer the non-redundant functionality. Our studies reveal that the highly similar histone isoforms can bring about changes in cell physiology by modulating chromatin dynamics.

## Results

### H2A1H/H2A1C expression level varies in cancer cell lines and amongst different normal tissues

**Fig. 1:**
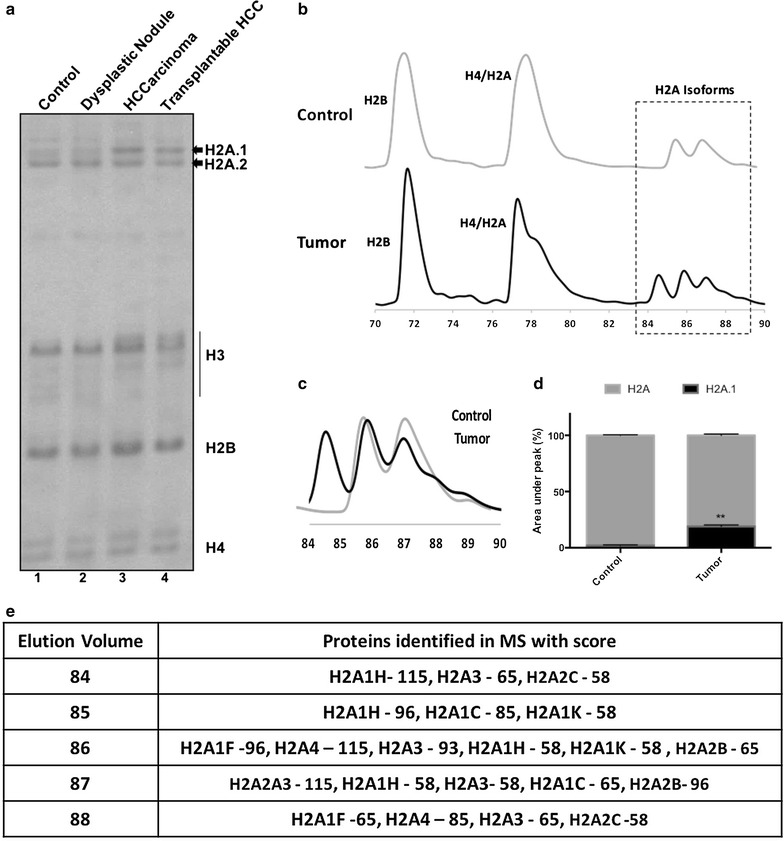
H2A.1 isoform, H2A1H’s expression predominantly increases during hepatocellular carcinoma. **a** AUT-PAGE analysis of histones (silver stained) isolated from xenograft tumor (marked transplantable HCC) along with histones from different stages of NDEA-induced liver cancer in rat. **b** RP-HPLC chromatogram of histones isolated from control (normal) and tumor tissues of rat liver. **c** Overlay of RP-HPLC profiles of histones isolated from control and tumor tissues of rat liver to depict major differences observed in the H2A region. **d** Bar graph depicting the area under the curve in the chromatogram for H2A isoforms. The total area under the curve was measured and considered as 100%. Then, the intensity of the peak at 84–85 ml (H2A.1, as per observed difference in mobility on AUT-PAGE) was measured and plotted as percentage of the total area. Error bar represents SEM of three independent experiments. **e** Table listing the H2A isoforms identified (with protein scores) in MASCOT search performed after mass spectrometry of the respective fractions of RP-HPLC. Scores greater than 52 are significant (*p* < 0.05). For the list of unique peptides identified, please see Additional file 1: Figure S3

Previously, we have reported the upregulation of H2A.1 isoforms during the progress of hepatocellular carcinoma (HCC) [9]. During the course of development of HCC, the animals were under the administration of NDEA. We wanted to see whether the increased expression of H2A.1 persists even without the influence of NDEA. To address this, a tumor was developed in the liver of Sprague–Dawley rat by feeding NDEA with drinking water. After the development of the tumor (105 days since the start of NDEA administration), a 3-mm^2^ tumor tissue was excised and subcutaneously implanted in a NOD-SCID mice. The NOD-SCID mice were not fed with NDEA. Two weeks post-implantation, the animals were sacrificed and the developed tumor was excised. Analysis of the isolated histones from the tumor resolved onto AUT-PAGE showed a higher expression of H2A.1 isoforms compared to the normal liver (Fig. 1a). This suggests that H2A.1 upregulation is indeed a stable alteration that occurs during the process of tumorigenesis. The changes in H2A composition in HCC were further appreciated by performing reverse-phase HPLC of the extracted histones (Fig. 1b) (see Additional file 1: Figure S2 for the complete elution profile). The most prominent difference in the chromatogram of the control vs tumor histones is the distinct peak at around 84 ml elution volume (Fig. 1c, d). Mass spectrometry followed by peptide fingerprinting of the eluted fractions 84 and 85 ml revealed high scores for the H2A.1 isoforms (H2A1H, H2A3, H2A1C, H2A1K) with the maximum score obtained for H2A1H (Fig. 1e) (see Additional file 1: Figure S3 for the peptides detected in MS). We next performed real-time PCR to check the transcript levels of the histone isoforms in normal vs tumor liver tissues (see Additional file 1: Figure S4). We performed normalization to the widely used normalization control, glyceraldehyde phosphate dehydrogenase (GAPDH) gene (see Additional file 1: Figure S4a). The histone isoforms are synthesized during the S-phase of the cell cycle. As the cells in tumor tissues are more proliferative, to normalize for the overall changes in the histone content, we also performed normalization to histone H4 genes (see Additional file 1: Figure S4b). The primers for H4 genes were designed to pick up all the H4 transcripts. Irrespective of the normalization control used, we found that H2A1H was the most prominently upregulated H2A isoform.

**Fig. 2:**
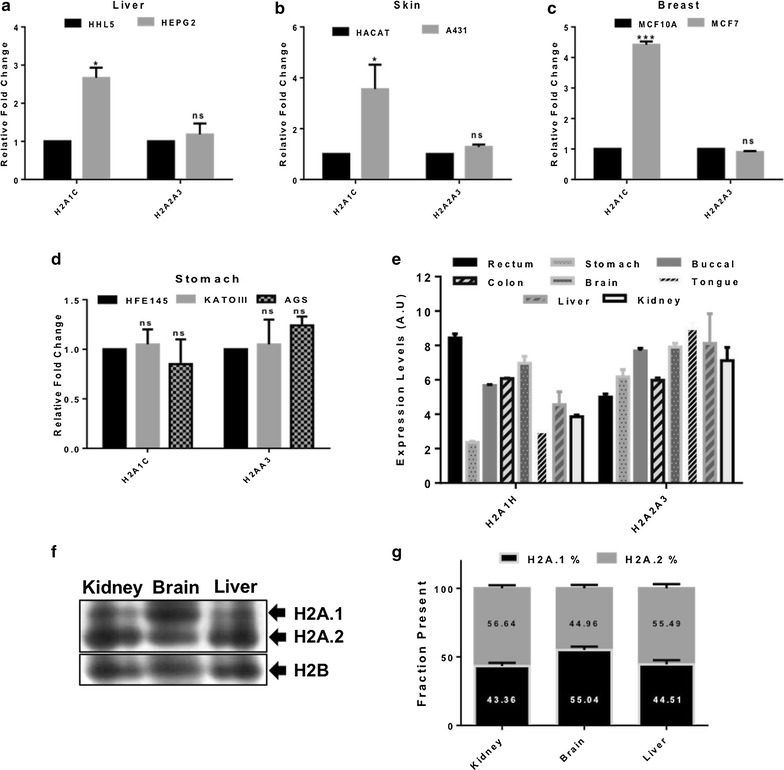
H2A.1/H2A1C expression varies drastically in different contexts. **a**–**d** Quantitative real-time PCR data showing the relative expression levels of H2A1C and H2A2A3 in different human cell lines (see text for more details). Error bar represents SEM of three independent experiments. **e** Graph showing the relative levels of H2A1H and H2A2A3 in various normal rat tissues, monitored at transcript level normalized to GAPDH by qRT-PCR. Error bar represents SEM of three independent experiments. **f** H2A and H2B region of AUT-PAGE analysis of histones (silver stained) isolated from the normal kidney, brain and liver tissues. **g** Quantitative analysis of the isoforms enrichment in the chromatin. Quantification of bands of H2A.1 and H2A.2 was performed by using the software GelAnalyzer. Normalization was done with respect to H2B as it appears as a single discrete band on AUT-PAGE. The data were plotted after taking the densitometric readings of three independent experiments. Error bars represent SEM of three independent experiments

In terms of the protein sequence, H2A1C in humans is the most similar to H2A1H of rat, differing in only the S16T substitution [see Additional file 1: Figure S7(c)]. The altered expression level of H2A1C has been reported in human cancers [10–13, 16–18]. Our observations in the rat hepatocellular carcinoma prompted us to investigate the expression level of H2A1C isoform in human transformed cell lines of the liver (HEPG2). We also included cell lines of skin (A431) and stomach (KATOIII, AGS) origin and their non-transformed immortalized counterparts, that is, HHL5 (liver), HACAT (skin) and HFE145 (stomach) in our study, as the expression level of H2A1C in these cell lines has not been previously reported. An increase in the relative expression of H2A1C was observed in HEPG2 and A431 (Fig. 2a, b). We did not find any significant changes in the levels of the isoform H2A2A3 (identical to rat H2A2A3). The two isoforms did not show any significant alteration in expression in both the transformed cell lines of the stomach with respect to their immortalized counterpart, that is, HFE145 (Fig. 2d). We also found upregulation of H2A1C in MCF7 consistent with a previously published report (Fig. 2c) [17].

We speculated that if H2A1H has some specific non-redundant function, then its expression may vary in different tissues. To test this hypothesis, the transcript level of H2A1H in different organs was compared. Marked variation in H2A1H level was observed. A very high level of H2A1H expression was observed in rectum (Fig. 2e). On the other hand, in the stomach and tongue tissues, the expression level was found to be particularly low (Fig. 2e). Isoform H2A2A3 exhibited much lesser variation in expression level (Fig. 2e). AUT-PAGE analysis of histones isolated from kidney, brain and liver demonstrates that the variations observed in transcript level of the H2A.1 isoform H2A1H is also reflected in protein expression (Fig. 2f). The brain showed an increased proportion of H2A.1, whereas kidney and liver have higher levels of H2A.2 isoforms (Fig. 2g).

### H2A1H isoform is functionally non-redundant from the H2A2A3 isoform

**Fig. 3:**
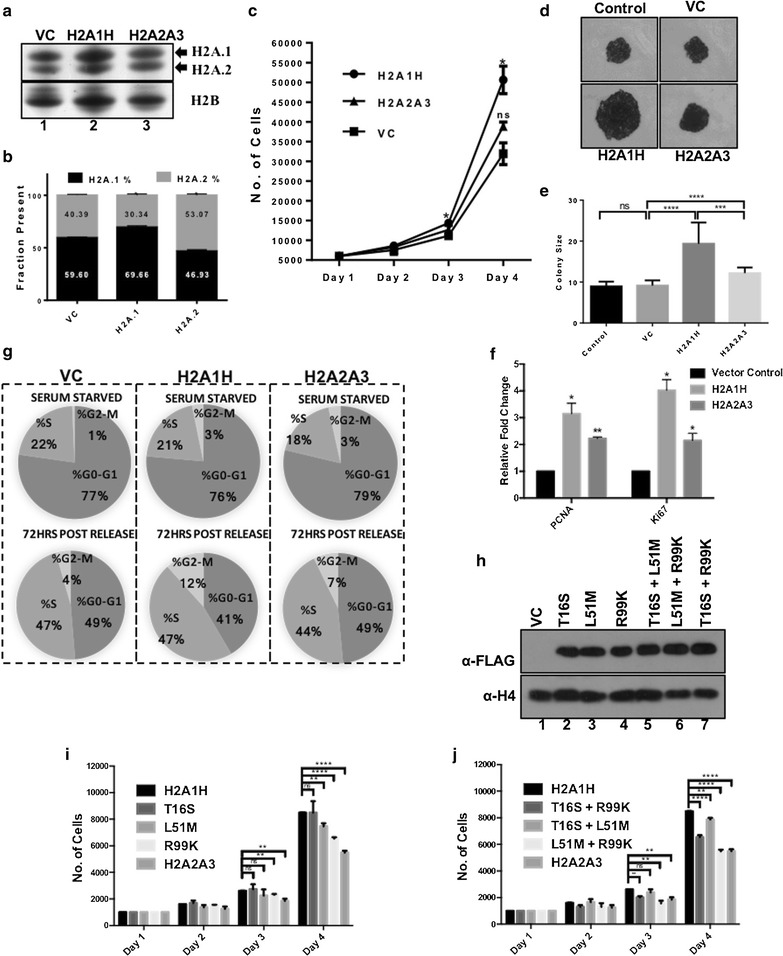
H2A1H overexpression leads to increase in cell proliferation. **a** AUT-PAGE analysis (silver stained) showing the enrichment of the H2A isoforms in chromatin upon their overexpression in CL38 cells. **b** Quantitative analysis of the isoforms enrichment in the chromatin. Quantification of bands of H2A.1 and H2A.2 was performed by using the software GelAnalyzer. Normalization was done with respect to H2B as it appears as a single discrete band on AUT-PAGE. The data were plotted after taking the densitometric readings of three independent experiments. Error bars represent SEM of three independent experiments. **c** Cell proliferation curves by MTT assay of H2A1H and H2A2A3 overexpressing CL38 cells in comparison with control CL38 cells. Error bars represent SEM of six independent experiments. **d** Colony formation assay of CL38 cells upon H2A1H and H2A2A3 overexpression. **e** Quantitative analysis of the colony sizes of 20 colonies each performed using ImageJ. Error bar represents SEM. **f** qRT-PCR for the cell proliferation markers Ki67 and PCNA on H2A1H and H2A2A3 overexpression normalized to 18S rRNA. Error bars represent SEM of three independent experiments. **g** Cell cycle analysis of the CL38 cells exogenously overexpressing H2A isoform post-serum starvation and release. **h** The expression level analysis of the CL38 cells expressing H2A1H single or double mutants with anti-FLAG antibody. **i** Bar graph depicting the proliferation of the CL38 cells expressing H2A1H single mutants by the MTT assay. Error bars represent SEM of 6 independent experiments. **j** Bar graph depicting the proliferation of the CL38 cells expressing H2A1H double mutants by MTT assay. Error bars represent SEM of six independent experiments. VC—vector control. H2A1H, H2A2A3 and their mutants in the figure are the genes cloned and expressed as FLAG tagged proteins in pcDNA3.1(+) vector

The expression level of H2A.1 isoforms varies in different tissues, differentiation status, age and diseases. Based on our results, we wanted to test the effect of overexpressing H2A.1 isoform H2A1H on cell physiology. Two cell lines that are derived from the liver of NDEA-administered Sprague–Dawley rats were chosen for our studies: CL44 (pre-neoplastic), with an equimolar ratio of H2A.1 and H2A.2, and CL38 (neoplastic), in which H2A.1 is naturally elevated (see Additional file 1: Figure S5). By RT-PCR, we validated that the CL38 cells express higher levels of the H2A1H isoform. Localization of YFP-tagged H2A1H/H2A2A3 in CL38 cells suggested that both the isoforms are incorporated across the entire chromatin (see Additional file 1: Figure S5). By isolating histones from the CL38 cells exogenously overexpressing the isoforms [pcDNA3.1(+) vector] and resolving them on AUT-PAGE, we confirmed that the overexpression of H2A1H leads to its increased abundance in the chromatin (Fig. 3a, b).

A marked increase in proliferation was observed in the CL38 cells on exogenous overexpression of H2A1H (Fig. 3c). Similar effects were reflected in the colony formation assay, with H2A1H overexpressing colonies substantially larger (Fig. 3d, e). Associated upregulation in proliferation markers Ki67 and PCNA was also noted by qRT-PCR (Fig. 3f). To see the effect of the isoforms overexpression on the cycling of cells, we studied the cell cycle profile of G1-enriched H2A1H/H2A2A3 CL38 cells post-72-h serum release. Overexpression of H2A1H led to a discernible increase in the mitotic cell population (12%) compared to the vector control (4%) (Fig. 3g). We also observed an increase in the mitotic cell population with H2A2A3 overexpression (7%) compared to the vector control (4%). This was also reflected in the proliferation assays (Fig. 3c, d). No significant difference in the closure of the wound in scratch assays performed with CL38 cells on H2A1H overexpression was perceived (see Additional file 1: Figure S6a) in comparison with H2A2A3 overexpression. Notably, we did not observe any significant change in the proliferation of CL44 cells upon H2A1H overexpression (see Additional file 1: Figure S6b). Importantly, during liver regeneration post-partial hepatotectomy, H2A.1 expression was not found to alter [8]. Taken together, these data suggest that although H2A1H expression provides a growth advantage to cells, its expression is not always correlated with proliferation (discussed later).

### Leu51 and Arg99 are important in conferring the non-redundant functionality to the H2A1H isoform

The H2A isoforms, H2A1H and H2A2A3, differ in three residues in their primary amino acid sequence (see Additional file 1: Figure S7a). To understand which residue(s) are important for the non-redundant functionality of H2A1H, we substituted the residues of H2A1H to the corresponding ones of H2A2A3. MTT assays performed with CL38 cells suggested that mutating R99K of H2A1H drastically reduced the pro-proliferative effect observed on its overexpression (Fig. 3i). Mutating L51M also negatively affected cell proliferation and had a synergistic effect when substituted alongside R99K (Fig. 3j). The assays were conducted with populations showing similar levels of overexpressed proteins to rule out possible variations resulting from any differences in the expression level (Fig. 3h). Notably, the 16th residue where the rat H2A1H and human H2A1C differ did not have any significant effect on the non-redundant effects of H2A1H in the assays performed by us (Fig. 3i, j).

### Leu51 and Arg99 of H2A1H are present at important locations in the nucleosome and may potentially impact its stability

Our results show that the expression of H2A1H varies markedly in different states and it does have non-redundant functionality. Further, Leu51 and Arg99 contribute majorly in conferring the non-redundant functionality to the H2A1H isoform. We next wanted to address how the H2A1H isoform imparts its non-redundant functional effects.

We carried out in silico simulation of mononucleosome and looked for the interactions of the three differential residues between H2A1H and H2A2A3 in the nucleosome core particle (NCP). The 16th residue of H2A is involved in interactions with the minor groove of DNA in the NCP, residue 51st lies in the dimer interface with H2B, and residue 99th of H2A interacts with the H4 tail in the octamer core (see Additional file 1: Figure S7b). Therefore, potentially the alterations at these residues can alter the stability of the nucleosome and its subcomplexes.

### The H2A1H-H2B dimer is less stable than the H2A2A3-H2B dimer

**Fig. 4:**
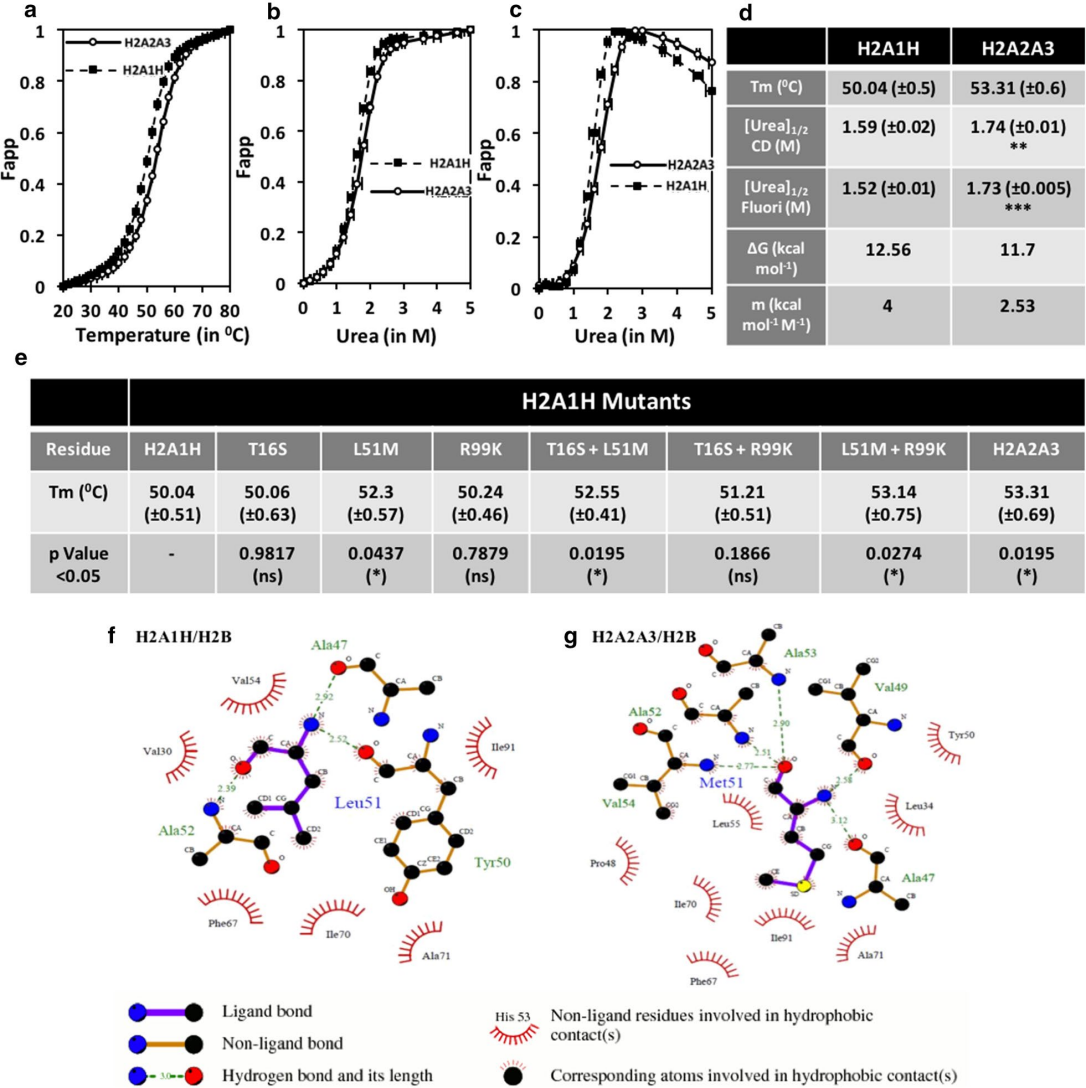
H2A1H-H2B dimer is less stable than the H2A2A3-H2B dimer. **a**, **b** Apparent fraction unfolded (Fapp) obtained from the analysis of the CD spectra of H2A1H-H2B and H2A2A3-H2B monitored during thermal and urea denaturation. **c** Fapp obtained from the analysis of the fluorescence spectra of H2A1H-H2B and H2A2A3-H2B monitored during urea denaturation. Error bar represents SEM of six independent experiments. **d** Comparative determination of the various parameters obtained by the CD and fluorescence spectra of H2A1H-H2B and H2A2A3-H2B. **e** Comparison of temperature of melting (Tm) of various H2A1H single, double mutants and H2A2A3 with H2A1H. **f**, **g** Ligplots depicting the interaction of the 51st residue of both H2A1H and H2A2A3 in the H2A–H2B dimer interface

To investigate the possibility discussed above, we compared the in vitro stability of the H2A1H-H2B with the H2A2A3-H2B dimer reconstituted using purified recombinant histones. Equilibrium unfolding of the reconstituted full-length H2A–H2B dimers, which was previously described [19], was used to perform the stability analysis. For details pertaining to the structural and stability characterization of the dimers, please refer to the “Methods” section. Once the equilibrium unfolding curves for both H2A1H-H2B and H2A2A3-H2B dimers were obtained, a comparative analysis of their stability was carried out (Fig. 4a). Co-plotting the Fapp (apparent fraction unfolded) of the H2A1H-H2B and H2A2A3-H2B dimers against the increasing temperature/denaturant concentration shows a hysteresis, suggestive of the difference in the propensity to unfold in response to the denaturant (Fig. 4a–c). The temperature of melting (Tm) for the H2A1H-H2B dimer was determined to be 50.04 °C, whereas that of the H2A2A3-H2B dimer was found to be higher by ~ 3 at 53.31 °C (Fig. 4d), suggesting that the former is less stable. The circular dichroism (CD) and the fluorescence data plotted in response to the increasing chemical denaturant concentration were in good agreement with each other. The [urea]_1/2_ for the H2A1H-H2B dimer was obtained as 1.59 and 1.52 M, respectively, using the two methods. The [urea]_1/2_ for the H2A2A3-H2B dimer was found to be 1.74 and 1.73 M with CD and fluorescence spectroscopy, respectively. Further, the *m* value obtained for the H2A1H-H2B dimer was 4 kcal mol^−1^ M^−1^ and that for the H2A2A3-H2B dimer was 2.53 kcal mol^−1^ M^−1^ (Fig. 4d) which are suggestive of the higher sensitivity of the H2A1H-H2B dimer to the denaturant concentration.

### The L51M substitution in H2A at the dimer interface with H2B is primarily responsible for the differential stability

The stability of the H2A1H-H2B dimer was determined to be lower than of the H2A2A3-H2B dimer. Subsequently, the effect of mutating the three residues in which the two H2A isoforms differ was investigated on the dimer stability by carrying out thermal denaturation with the reconstituted mutant dimers. Studies with the mutants suggest that the L51M alteration had the biggest impact on the stability of the dimers (Fig. 4e). Mutating L51M in H2A1H increased the Tm from 50.04 to 52.3 °C and that of H2A2A3 to M51L (H2A1H T16S + R99K) decreased the stability by 2.1 °C (Fig. 4e).

Leucine-to-methionine alteration at the 51st residue, which we found to be primarily responsible for the differential stability, has been suggested to be context dependent [20]. Although the van der Waals volume occupied by leucine is the same as for methionine, two opposing forces are at play when leucine-to-methionine substitution occurs. The substitution of methionine with leucine within the interior of a protein is expected to increase the stability because of both a more favorable solvent transfer term and the reduced entropic cost of holding the leucine side chain in a defined position. At the same time, this expected beneficial effect may be offset by steric factors due to the differences in the shape of leucine and methionine [20]. To understand the possible alteration in interactions on the incorporation of methionine, we carried out energy minimization of the structures. As depicted in the ligplots, the substitution L51M led to an increased number of hydrogen bonds and hydrophobic interactions that explains the higher stability observed in denaturation experiments (Fig. 4f, g). Altering the 16th and 99th residues in isolation did not have a major effect on the dimer stability; however, mutating R99K along with L51M had a synergistic effect on stabilizing the dimer by an additional increment in stability by ~ 0.8 °C (discussed in more detail in “Discussion” section).

### The H2A1H isoform-containing nucleosomes are more stable owing to the formation of higher number of hydrogen bonds

**Fig. 5:**
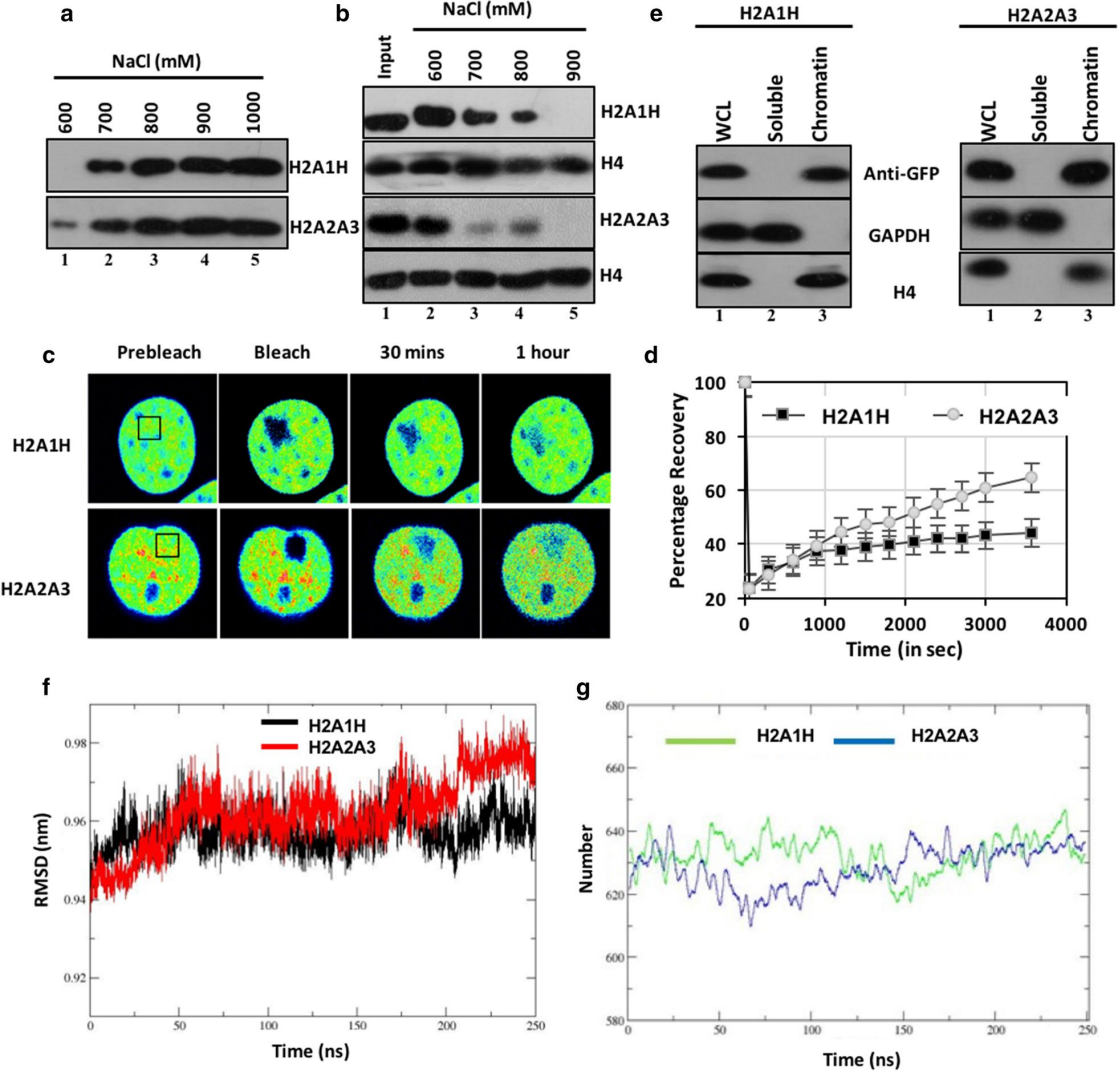
H2A1H-containing nucleosome is more stable than the H2A2A3 nucleosome. **a**, **b** Levels of H2A1H and H2A2A3 in the soluble and chromatin fractions upon incubation of CL38 cells in buffers of increasing ionic strength. **c** FRAP assay performed with CL38 cells expressing YFP-tagged H2A1H and H2A2A3. Recovery was monitored for a period of 1 h. **d** Graph depicting the percentage recovery of YFP-H2A1H and YFP-H2A2A3 over a span of 4000 s. Error bar represents SEM of ten independent experiments. **e** Cellular fractionation of the CL38 cells followed by immunoblotting with the marked antibodies to determine the distribution of histones. **f** RMSD of H2A1H and H2A2A3 nucleosomes over a span of 250 ns of molecular dynamic simulation. **g** Hydrogen bond analysis of the H2A1H- and H2A2A3-containing nucleosome over the span of 250 ns of molecular dynamic simulation (MDS)

To understand the importance of the alteration in dimer stability in the context of the chromatin, we investigated the effect of the incorporation of these isoforms on the nucleosome stability. Beyond 600 mM NaCl concentration, the nucleosome core particle starts losing its integrity as the histone H2A–H2B dimers start to irreversibly dissociate from the particle [21]. Hence, to compare the stability of the chromatin association of H2A1H-H2B and H2A2A3-H2B dimer, the chromatin was incubated in buffers of increasing ionic strength starting from 600 mM NaCl. Detectable levels of the H2A2A3 isoform (FLAG tagged) were obtained in the soluble fraction (supernatant post-centrifugation at 13,000*g* for 30 min, 4 °C) at a lower ionic strength (600 mM NaCl) compared to H2A1H (700 mM NaCl) (Fig. 5a). Analysis of the chromatin fraction also indicated that the H2A1H isoform is more resistant to elution from the chromatin with increasing ionic strength compared to the H2A2A3 isoform (Fig. 5b).

To see whether the more stable association of H2A1H with chromatin is also reflected in its dynamics, we monitored the recovery of fluorescently tagged histone isoforms in a bleached region of the nucleus of CL38 cells (Fig. 5c). We documented that the distribution of both the isoforms is similar in the soluble and chromatin-bound fractions with undetectable levels in the soluble fraction (Fig. 5e). The percentage recovery of H2A1H after 1 h was markedly less (44.14%) compared to H2A2A3 (64.7%) (Fig. 5c, d) in the FRAP assay, suggesting that H2A1H is less dynamic than the H2A2A3 isoform.

To understand the basis of the increased stability of H2A1H-containing nucleosomes, we performed the molecular dynamic simulation (MDS). The convergence of the MD simulation in terms of the structure was calculated by the root mean square deviation (RMSD) with respect to the initial structure. The RMSD analysis was in agreement with the in vitro data with lower RMSD of H2A1H-containing system, suggesting that it forms more stable nucleosomes as compared to H2A2A3 (Fig. 5f). Corroboratively, the hydrogen bonding analysis shows that during the course of the simulation, H2A1H nucleosome has a higher number of hydrogen bonds (Fig. 5g). The RMSD of the octamer and DNA independently showed a similar trend (see Additional file 1: Figure S10).

### Leu51 and Arg99 residues lead to the increased stability of H2A1H-containing nucleosomes as compared to H2A2A3-containing ones

**Fig. 6:**
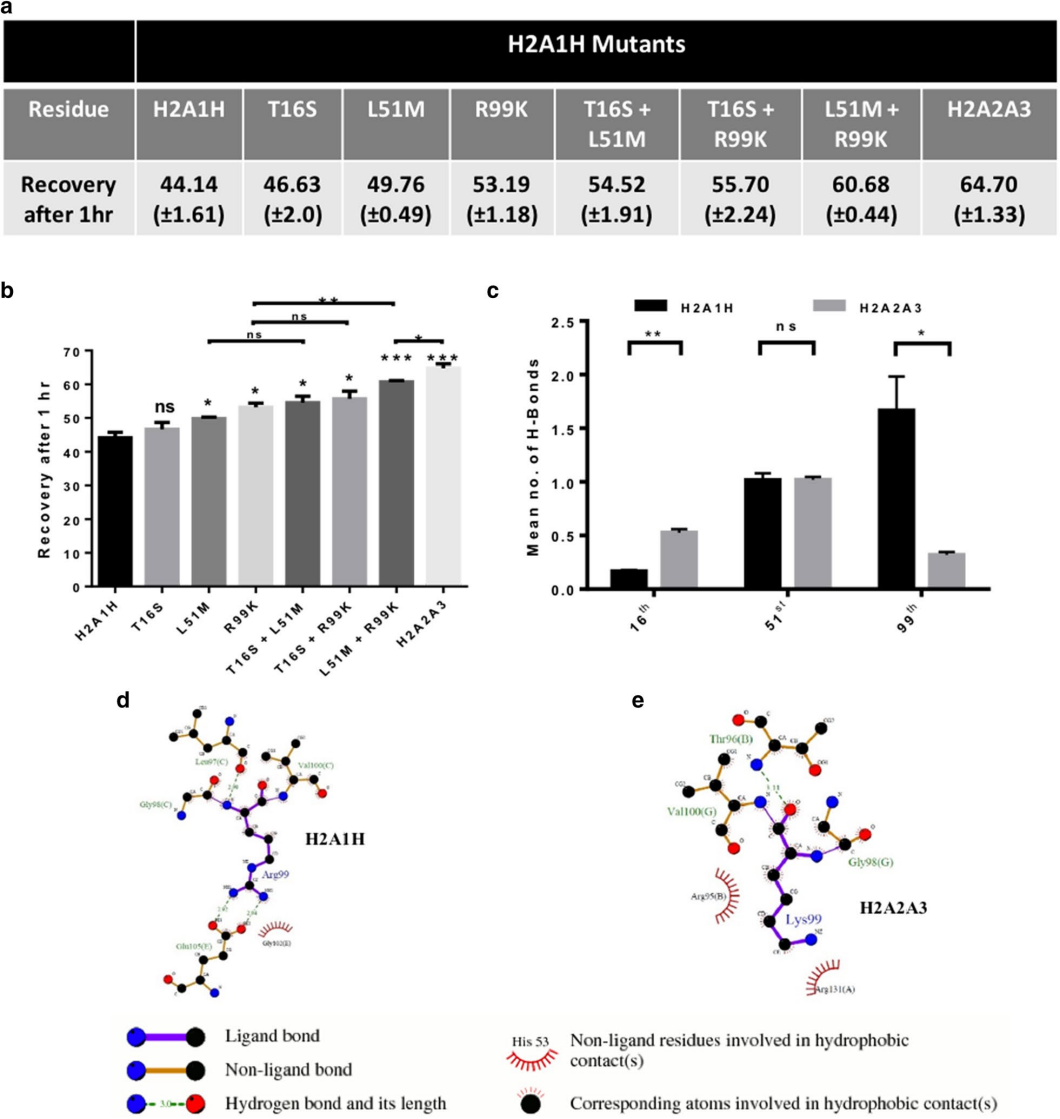
K99R alteration makes the H2A1H-containing nucleosomes more stable. **a**, **b** Comparative analysis to determine the percentage of recovery after photo bleaching, for 1 h amongst various H2A1H single, double mutants and H2A2A3 with H2A1H. Error bar represents SEM of ten independent experiments. **c** Comparative determination of the hydrogen bonds formed by three differential residues (16th, 51st and 99th) with their neighboring residues in H2A1H and H2A2A3 during MDS of nucleosomes. Error bar represents SEM of three independent experiments. **d**, **e** Ligplot depicting the hydrogen and hydrophobic interactions of the 99th residue of (**d**) H2A1H and **e** H2AA3 with the neighboring residues

We carried out site-directed mutagenesis of the isoforms followed by FRAP in CL38 cells to identify the important alteration(s) that is majorly responsible for the difference in chromatin dynamics of H2A1H and H2A2A3. The R99K substitution, which is involved in the interaction with the H4 tails in the NCP, independently brought about the most drastic increase (20%) in the dynamics of H2A1H followed by L51M (12%) (Fig. 6a, b). Mutating both the L51M and R99K together led to almost similar dynamics as observed for H2A2A3. Mutating only T16S did not have a significant impact on the H2A1H dynamics. However, a synergism was observed when residue T16S was mutated alongside L51M and R99K (discussed later).

We wanted to understand how the substitutions with very similar amino acids brought about the observed changes in the nucleosome stability. Analysis of the number of hydrogen bonds formed by the residues at the three positions with nearby residues throughout the simulation time of 250 ns was performed for both the H2A1H- and H2A2A3-containing nucleosomes. The data suggested that the 51st and the 99th residues majorly participate in the formation of hydrogen bonds with very less contribution from the 16th residue (Fig. 6c). Importantly, the arginine at 99th position in H2A1H system forms more number of hydrogen bonds than lysine (Fig. 6c). The ligplots depicts the hydrogen and hydrophobic interactions between the 99th and nearby residues of H2A1H (Fig. 6d) and H2A2A3 systems (Fig. 6e).

### Principal component analysis suggests that H2A1H-containing nucleosome structures are better correlated

**Fig. 7:**
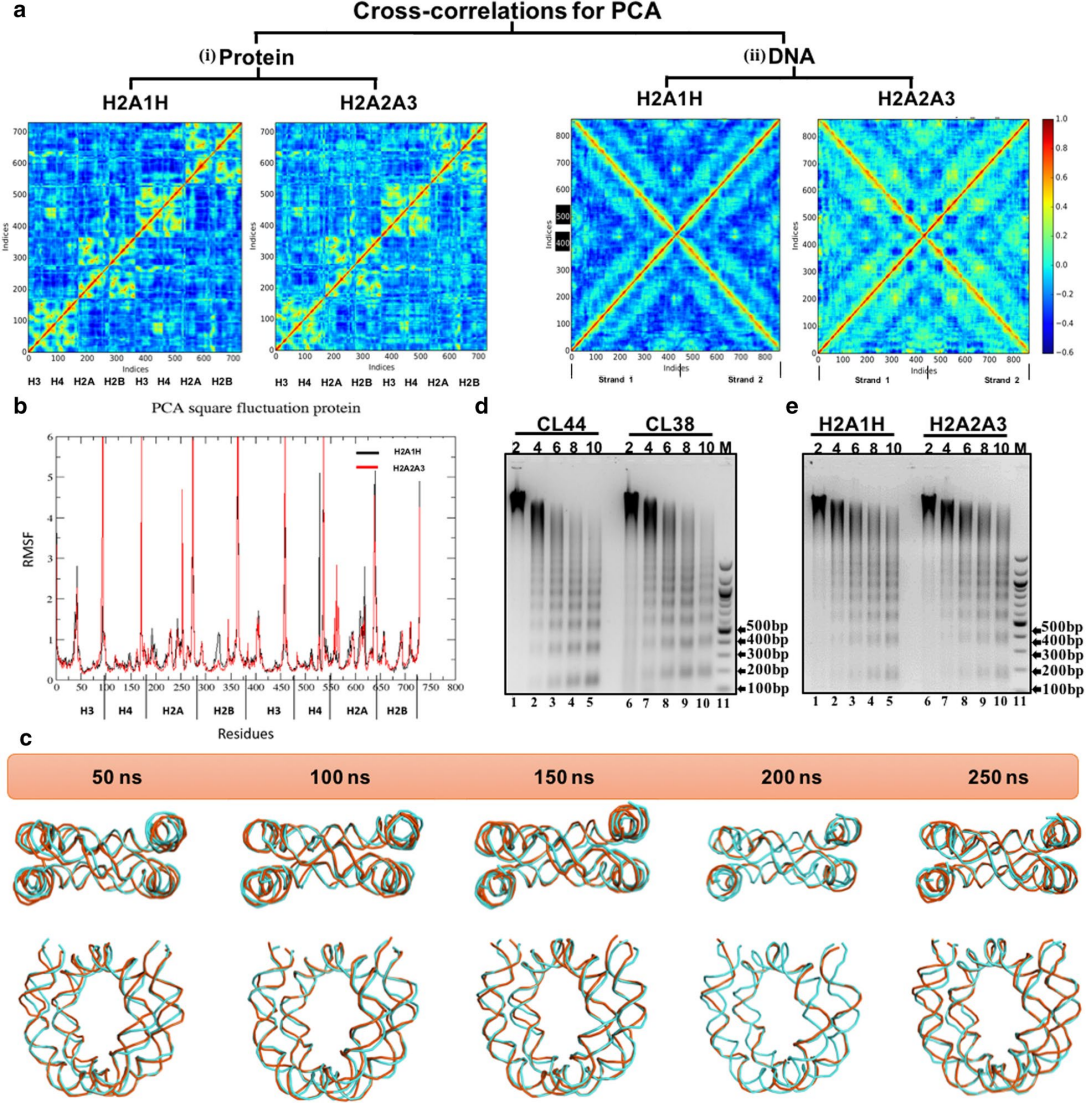
Principle component analysis (PCA) of H2A1H-containing nucleosomes is better correlated than of H2A2A3 with no changes in global structural chromatin organization in vivo. **a** (i, ii) Cross-correlation plots determining the atomic fluctuations at the protein and DNA level for H2A1H- and H2A2A3-containing nucleosome. The blue color indicates negative cross-correlation, while red color indicates positive cross-correlation. **b** Comparison of the PCA square fluctuations of DNA/protein amongst H2A1H- and H2A2A3-containing nucleosome. **c** Overlaid images of the nucleosomal DNA strands of histone H2A1H (green) and H2A2A3 (orange) isoform-containing systems at different time points during the course of simulation. The time points are indicated. **d**, **e** The accessibility of chromatin was monitored by performing micrococcal nuclease digestion assay and loading samples from the reaction at different time points on a 1.8% agarose gel. The DNA was visualized by EtBr staining. In figure **d**, samples from digested MNase-digested nuclei of the CL44 and CL38 cell lines at various time points were loaded. In figure **e**, samples from MNase-digested nuclei of ectopically overexpressing H2A1H and H2A2A3 CL38 cells were loaded

Next, the principal component analysis (PCA) was carried out to discriminate between relevant conformational changes in the protein structure from the background atomic fluctuations. The Fig. 7a(i) shows the cross-correlation plot for protein octamer for H2A1H and H2A2A3. In the H2A1H nucleosome, nearby interacting chains show a positive correlation, while the distant regions are showing a negative correlation. Generally, a positive correlation is seen in nearby residues with a synchronous motion, whereas a negative correlation is observed between distantly interacting residues with asynchronous motion. Histones H3 and H4 together form a dimer; therefore, H3 shows a positive correlation for H4, while negative correlation for rest of the histone chains. Similarly, H2A shows a positive correlation for H2B. The pattern of correlation observed with H2A1H- or H2A2A3-containing nucleosome is the same for nearby chains; however, the correlation between H2A2A3 and H2B (system 2) is slightly less positive compared to H2A1H- and H2B-containing nucleosome (system 1). Also, in system 2 there is a less negative correlation between distant chains. Thus, comparing the cross-correlation data with the PCA square fluctuation (Fig. 7b) it can be seen that the negatively correlated motion between distant chains is providing a rigidity and stability to the H2A1H nucleosome. The cross-correlation of DNA [Fig. 7a(ii)] follows the same trend.

### Incorporation of the H2A1H isoform does not impart structural alterations to the chromatin

The difference in the cross-correlation plot for the DNA of H2A1H- and H2A2A3-containing systems [Fig. 7a(ii)] prompted us to investigate whether there might be a structural alteration in the DNA on the incorporation of the H2A isoforms. Overlaying the structures of different time points of simulation suggested that there is no prominent structural alteration (Fig. 7c). To see whether there are any global changes in nucleosome spacing or chromatin accessibility, the chromatin of CL44 and CL38 cells was subjected to micrococcal nuclease (MNase) digestion. No structural alterations were discernible on resolving the digestion products on an agarose gel (Fig. 7d). Similarly, the digestion profile was virtually identical for the chromatins isolated from CL38 cells exogenously overexpressing the H2A1H/H2A2A3 isoforms (Fig. 7e), suggesting that the global chromatin structure and accessibility do not alter significantly on the incorporation of the H2A1H and H2A2A3 isoforms. However, more sensitive experiments are needed to be performed to rule out the possibility of very minute changes that might occur on the incorporation of the histone isoforms.

## Discussion

The non-redundancy of the histone isoforms has made the understanding of the epigenetic regulations employed by cells more complex, nevertheless, interesting. Previous studies have attempted to elucidate the role of the H2A1C isoform in context of cancer [17]; however, insights into the basic non-redundant role of H2A isoforms, which may contribute to the attainment or persistence of a particular physiological or pathological state, remain poorly addressed. Earlier we had reported that the expression of H2A.1 isoforms increases in HCC [9]. Considering the growing identification of a variety of H2A isoforms, we validated our earlier findings with the help of RP-HPLC. Further, we addressed the molecular basis of the functional non-redundancy of the histone H2A isoform H2A1H that is overexpressed in cancer.

We found the L51M alteration to have the most significant impact on the H2A–H2B dimer stability. The difference observed between the H2A1H-H2B and H2A2A3-H2B dimer stability is subtle compared to the change brought about by histone variants like H2A.Z [19]. This is consistent with previous reports where L to M replacements altered the protein stability only by 0.4–1.9 kcal/mol [22]. Possibly, the ubiquitous abundance of H2A isoforms in the genome, as opposed to variants, makes this difference significant to induce alterations in epigenetic regulation. Probably, the cell uses the histone variants to bring about major changes in gene regulation and has evolved the histone isoforms for subtle modulations of chromatin-mediated processes.

Interestingly, besides the involvement of the L51M alteration in determining H2A–H2B dimer stability, a synergistic effect was seen when residue R99K was mutated along with L51M. This was intriguing as the residue 99th is not present in the dimer interface. Arginine (in H2A1H) and lysine (in H2A2A3) are positively charged residues and play important roles in stabilizing proteins by forming ionic interactions and hydrogen bonds in the protein as well as with water [23]. Notably, the guanidinium group in arginine allows interactions in three possible directions through its three asymmetrical nitrogen atoms in contrast to only one direction of interaction allowed for lysine. Owing to this difference in geometry of the two amino acids, arginine might have a more stabilizing effect on proteins over lysine [20]. The presence of arginine in H2A1H probably stabilizes the H2A monomer more as compared to lysine in H2A2A3 which thermodynamically makes the H2A1H-H2B dimer less stable. Further, the ability of arginine to form a higher number of H-bonds compared to lysine is also reflected in our FRAP assay and MDS studies.

As discussed earlier, the altered stability of the H2A–H2B dimer will have its implications in the nucleosome stability. Previous MDS studies focussed on the histone octamer–DNA interactions revealed that the H2A–H2B dimer is the least stable part of the nucleosome and could make a significant contribution to the histone–DNA interaction dynamics [24]. We found that the H2A1H isoform gives rise to a more stable nucleosome although the H2A1H-H2B dimers were less stable. This is consistent thermodynamically as a less stable dimer would favor a more stable nucleosome. This is because the association of the H2A–H2B dimers with the nucleosome core particle (NCP) is a dynamic process. Therefore, there is an important equilibrium between the fully assembled NCP and partially unfolded NCPs in which the H2A–H2B dimers are less tightly bound or completely dissociated. The shift in this equilibrium will be affected by the overall entropy of the system, which, in turn, would depend on the free energy of the dissociated dimers. Therefore, the stability of the free H2A–H2B dimer will have consequences on the state of nucleosome assembly and its stability. A more stable H2A–H2B dimer should favor a more unfolded, dissociated state of the NCP. Similar to our observations, for the H2A.Z variant it was reported that the H2A.Z–H2B dimer was unstable as compared to the canonical H2A–H2B [19]; however, the nucleosome was found to be more stable [25].

A more stable nucleosome is expected to cause hindrance to chromatin-mediated processes like transcription, replication and repair. Previously, the HAR domain of H2A, which comprises of the residues 16–20 of the N-terminal tail, has been implicated in transcriptional repression owing to its ability to govern nucleosome dynamics by interacting with the minor groove of DNA [26]. Although the HAR domain was initially identified in yeast, it was later shown to be important in humans as well [17]. In addition, the S16A substitution at the HAR domain was found to disrupt its repressive ability [17]. Our data shows that the S16T substitution does not significantly alter nucleosome dynamics by itself. However, a synergism is observed when this substitution is carried out alongside alteration at 51st and 99th residues. This suggests that probably the presence of a serine at the 16th position instead of threonine favors the disassembly of the H2A–H2B dimer from the NCP; however, the interactions of the 51st and 99th residue are predominant in governing the nucleosome stability.

One very important aspect that collectively emerges from our study and the earlier reports is that the functional effects exhibited by the H2A isoforms might be context dependent, in terms of both the extent and the effect itself. For example, the pro-proliferative effect conferred by H2A1H was not observed in the pre-neoplastic CL44 cells. Notably, the human H2A1C isoform, which was initially reported to be downregulated in CLL, was shown to exhibit anti-proliferative effects [10]. However, in a later study with a higher number of samples, H2A1C levels were found to be higher in CLL patients compared to the samples from healthy individuals [12]. Further, the high expression of H2A1H seen in the brain cells, which are terminally differentiated and do not regenerate, suggests that the actual functional effect of H2A1H may also be context dependent and need not be always proliferation associated. Interestingly, the higher expression of H2A1C has been seen in chemo-resistance in the pancreatic cancer cell lines [27]. It remains to be seen how overexpression of H2A1C might contribute to that.

Based on the discussion above, some of the questions that arise are: what determines the context in which the non-redundant functionality of the H2A isoforms is exhibited? And in those contexts, which are the genes that are regulated by a particular isoform? Difficulty in raising specific antibodies against the endogenous H2A1H and H2A2A3 proteins, which differ in only three residues that are well spaced apart, poses a technical challenge to address these questions. It is reasonable to hypothesize that other factors that contribute to the epigenetic landscape of cells and/or the differential PTMs that the histone isoform itself may undergo, determine the context in which the differential functional effects of the H2A isoforms are exhibited. Interestingly, Arg 99 of H2A has been shown to undergo methylation [28]. One study, which has tried to identify the genes in the particular context, shows that H2A1C isoform controls ER target genes in ER-positive breast cancer cell lines [17]. Interestingly, the deletion of the H2A N-terminal domain (Δ4-20) led to upregulation of only 248 genes [26]. Clearly, much remains to be understood of the correlation between H2A-mediated nucleosome stability and gene expression.

## Conclusion

H2A1H-containing nucleosomes are more stable owing to the M51L and K99R substitutions that also have the most prominent effect on cell proliferation, suggesting that the nucleosome stability is intimately linked with the physiological effects observed. Possibly, the increased nucleosome stability resulting from H2A1H incorporation contributes to the contextual alteration in the global gene expression pattern that collectively promotes the attainment of different physiological states. This possibility of the non-redundant function, when extended to the plethora of the histone isoforms (H2A, 12 isoforms; H2B, 16 isoforms; H3, 6 isoforms; and H1, 6 isoforms), truly increases the complexity of the epigenome by many folds. Undoubtedly, such complexity is the necessity for multicellular organisms as the diversity in the epigenome plays a central role in cell-type-specific gene expression. This, in turn, leads to the specialized functions in thousands of cell types with the same genome.

## Methods

### Antibodies and reagents

Anti-FLAG antibody (Sigma-Aldrich, A8592), anti-GFP antibody (Roche, 11,814,460,001), anti-GAPDH antibody (Ambion, AM4300), anti-H4 (Millipore, 07-108) and oligos (Sigma-Aldrich) were used.

### Animal handling and experiments

All the experiments were performed on male Sprague–Dawley rats (spp. *Rattus norvegicus*) or SCID mice after approval of the Institute Animal Ethics Committee, Advanced Centre for Treatment Research and Education in Cancer and the Committee for the Purpose of Control and Supervision on Animals, India, standards. Protocol to induce the sequential stages of liver carcinogenesis is as previously described [9].

### AUT-PAGE

Core histones were applied horizontally to the top of a 15% AUT-PAGE and sealed using sealing buffer (1% w/v agarose, 0.75 mol/L potassium acetate, pH 4, 20% v/v glycerol and 0.001% pyronin Y). The gel was electrophoresed at a constant voltage of 200 V.

### RP-HPLC

Reversed-phase separation was carried out on a C18 column (1.0 × 250 mm, 5 mm, 300 Å; Phenomenex). Mobile phases A and B consisted of water and acetonitrile, respectively, with 0.05% trifluoroacetic acid. The flow rate was 0.42 ml/min, and the gradient started at 20% B and increased linearly to 30% B in 2 min, to 35% B in 33 min, 55% B in 120 min and 95% B in 5 min. After washing with 95% B for 10 min, the column was equilibrated at 20% B for 30 min, and a blank was run between each sample injection.

### Mass spectrometry

Histone spots of interest from AUT-PAGE and the fractions of RP-HPLC were subjected to matrix-assisted laser desorption/ionization mass spectrometry (MALDI-MS) using MALDI-TOF/TOF mass spectrometer (Bruker Daltonics Ultraflex II). In brief, gel pieces were washed, destained, reduced, alkylated and subjected to in-gel digestion, and HPLC fractions were subjected to in solution trypsin digestion. Mass spectra were acquired on reflector ion positive mode. Database searching for protein masses was carried out using MASCOT search engine (version 2.2.03) by comparing the peptide masses with those in the NCBInr protein database (database version: NCBInr_20080812.fasta) in Rattus species. The searches were carried out with trypsin digestion, one missed cleavage, fixed carbamidomethylation of cysteine residues and optional oxidation of methionine with 100 ppm mass tolerance for monoisotopic peptide masses.

### Isolation of total RNA and PCR

Total RNA was extracted from cells as per the manufacturer’s (Macherey-Nagel) instructions. It was further treated with DNaseI for 30 min at 72 °C to degrade any possible DNA contamination. RNA (2 µg) was subjected to reverse transcription using M-MLV reverse transcriptase and random hexamer primers according to the manufacturer’s (Fermentas) instructions. cDNAs were then amplified with the corresponding gene-specific primer sets (see Additional file 1: Figure S11). For RT-PCR, PCR was conducted for 24 cycles using the condition of 30 s at 94 °C, 1 min at 58 °C and 1 min at 72 °C. The PCR products were analyzed on a 1% agarose gels containing 0.5 µg/ml ethidium bromide. For real-time PCR Syber-Green from Ambion was used. The reactions were performed and monitored using QuantStudio 12K Flex Real-Time PCR System.

### Histone purification and dimerization

Histones were purified and the H2A–H2B dimers were reconstituted as previously described [29]. The dimers were purified by size exclusion chromatography using HiLoad 16/60 Superdex-200 gel filtration column (GE).

### Equilibrium unfolding of dimers

The dimers were subjected to equilibrium unfolding which was monitored by observing both secondary and tertiary structure changes.

#### Secondary structure changes

Unfolding was observed in response to thermal and chemical denaturant by circular dichroism.

#### Thermal unfolding

Unfolding was carried out starting from 20 up to 80 °C with a 2 °C increment and an equilibration time of 3 min. The CD spectra of only three temperatures are plotted for clarity. Analysis of the thermal unfolding curves suggests that dip at 222 nm can serve as a good spectroscopic probe for monitoring secondary structure unfolding [see Additional file 1: Figure S8a(i)]. Further, the unfolding was completely reversible with no protein aggregation as suggested by the completely overlapping unfolding and refolding curves [see Additional file 1: Figure S8a(ii)]. The data obtained could be fit into two-state unfolding model for dimeric proteins with residual in the range of only ± 2 using IgorPro [see Additional file 1: Figure S8a(iii)].

#### Chemical unfolding

Urea-induced denaturation was also monitored with CD with an increment of 0.2 M urea concentration starting from 0 M, and like thermal denaturation, the dip at 222 nm in the CD spectra was used to plot the unfolding [see Additional file 1: Figure S8b(i)]. Initially, a titration up to 8 M urea was carried out; however, as the unfolding was complete in 3 M urea, subsequent titrations were performed with up to 5 M concentration of urea. The denaturation was completely reversible [see Additional file 1: Figure S8b(ii)]. Similar to the thermal unfolding data, the chemical denaturation data could be fit into the two-state unfolding model [see Additional file 1: Figure S8c(iii)].

#### Tertiary structure changes

To follow the tertiary structure unfolding, urea-induced denaturation monitored by fluorescence spectroscopy was performed.

#### Chemical unfolding

On carrying out urea-induced denaturation, there was a drop in the fluorescence intensity with the unfolding of proteins as expected because of the quenching of fluorescence of the tyrosines previously buried in the dimer interface [see Additional file 1: Figure S8c(i)]. The drop in the intensity of emission maxima at 305 nm could be used for monitoring and plotting denaturation as there was no apparent redshift [see Additional file 1: Figure S8c(i)]. The folding was reversible [see Additional file 1: Figure S8c(ii)]; however, the pre- and post-transition baselines in the urea denaturation curve had a positive slope as observed in previous reports [19]. However, to ensure that transitions were not missed during the unfolding process, denaturation was carried out with GdmCl as well. Similar pre- and post-transition baselines corroborated the urea denaturation data (see Additional file 1: Figure S9). The unfolding also showed a concentration dependence as is expected for a dimeric protein [see Additional file 1: Figure S8c(iii)] and could be fit into the two-state model of unfolding [see Additional file 1: Figure S8c(ii)] substantiating the data obtained for secondary structure unfolding.

##### Site-directed mutagenesis

For making mutants for the study, site-directed mutagenesis was performed using the kit and guidelines given in the QuickChange™ Site-Directed Mutagenesis Kit from Stratagene. Oligos were procured from Sigma-Aldrich.

##### Data fitting

The unfolding data were fit into the two-state model of unfolding as described previously [30].

##### FRAP assay

H2A1H and H2A2A3 coding sequences were cloned into peYFPn1 (YFP at C-terminal) vector and transfected in CL38 cells. LSM510 Meta (Zeiss) microscope equipped with CO_2_ and temperature maintenance accessories was used to carry out the studies. The nuclei was bleached (in a box of fixed area) using 488-nm laser set at 100% power, and the recovery in the region was monitored for 1 h. Images were taken at 30-s intervals for the first 15 min and then at a 5-min interval for the remaining 45 min to minimize photobleaching. Quantification of the recovery was done as described previously [31].

##### Molecular dynamics simulation

All the simulations were performed using the Gromacs-4.6.5 software, with periodic boundary conditions. The particle mesh Ewald method was used to treat the long-range electrostatics, together with a cutoff of 1.2 nm for the short-range repulsive and attractive dispersion interactions, which were modeled via a Lennard–Jones potential. The Settle algorithm was used to constrain bond lengths and angles of water molecules and the P-Lincs for all other bond lengths. The time step of 2 fs was used for the entire system. The temperature was kept constant at 300 K by using the Nose–Hoover thermostat method. To control the pressure at 1 atmosphere, Parrinello–Rahman method was used. The following DNA sequence was used to model nucleosomes: ATCAATATCCACCTGCAGATTCTACCAAAAGTGTATTTGGAAACTGCTCCATCAAAAGGCATGTTCAGCTGAATTCAGCTGAACATGCCTTTTGATGGAGCAGTTTCCAAATACACTTTTGGTAGAATCTGCAGGTGGATATTGAT.

##### Cell line maintenance and synchronization

The cells from the human origin were maintained in appropriate growth media depending on the line at 37 °C with 5% CO_2_ supplemented with 10% FBS, 100 U/ml penicillin, 100 mg/ml streptomycin and 2 mM l-glutamine (Sigma). Cell lines CL38 and CL44 from rat liver origin were cultured in MEM (invitrogen) media with 10% FBS and were maintained at 37 °C with 5% CO_2_.

For overexpression experiments, mammalian expression vectors with CMV promoters (pcDNA3.1, pcDNA3.1 FLAG HA or peYFPn1) were used. The coding sequence of H2A1H (NM_001315492.1) or H2A2A3 (NM_001315493.1) was cloned in frame. For generating stable lines, the CL38 and CL44 cells were transfected with vectors (empty or encoding gene of interest) using TurboFect (ThermoFisher). Stable populations were selected by adding G418 (Sigma-Aldrich) in the growth media.

For cell cycle experiments, cells were enriched in the early G1-phase by serum starvation (0.1% FBS) for 24 h. Cells were released from the arrest by supplementing the media with 10% FBS.

##### Cell cycle analysis

Ethanol-fixed cells were washed twice with PBS and suspended in 500 µl of PBS with 0.1% Triton X-100 and 100 µg/ml of RNaseA followed by incubation at 37 °C for 30 min. After incubation, propidium iodide (25 µg/ml) was added followed by incubation at 37 °C for 30 min. DNA content analysis was carried out in a FACSCalibur flow cytometer (BD Biosciences, USA). Cell cycle analysis was performed using the ModFit software from Verity house.

##### Histone isolation and immunoblot analysis

First, nuclei were isolated from cells. For this, the cell pellet was resuspended in 0.1 ml PBS in a microcentrifuge tube. To this suspension, 0.9 ml lysis solution (250 mM sucrose, 50 mM Tris–Cl pH 7.5, 25 mM KCl, 5 mM MgCl_2_, 0.2 mM PMSF, 50 mM NaHSO3, 45 mM sodium butyrate, 10 mM β-ME and 0.2% v/v Triton X-100) was added. Tube was inverted several times and centrifuged for 15 min at 800 g, 4 °C. For nuclei isolation from tissues, the tissue was homogenized in hypotonic buffer (10 mM HEPES pH 7.5, 10 mM KCl, 0.2 mM EDTA, 0.1% NP40, 10% glycerol, 1 mM DTT) using Dounce homogenizer. The homogenate was overlayed on the same buffer containing 1.8 M sucrose and ultracentrifuged (20,000*g* for 2 h). The nuclear pellet obtained was subjected to histone extraction by acid extraction method by adding 0.3 ml of 0.2 M H_2_SO_4_. The tubes were vortexed thoroughly with intermittent incubation on ice. The tubes were then centrifuged at 13,000*g*, 4 °C for 30 min. The supernatant was transferred to a fresh tube without disturbing the pellet. The proteins in the supernatant were precipitated by adding 4 volumes of acetone and stored overnight at −20 °C. The tubes were then centrifuged at 13,000*g*, 4 °C for 10 min. The pellet was washed once in chilled acidified acetone (0.05 M HCl in 100% acetone) and once in chilled 100% acetone. Protein pellet was dried in vacuum centrifuge for 15 min. The pellet was resuspended in 0.1% β-ME at −20 °C. For immunoblotting, histones were resolved on 18% SDS–polyacrylamide gel, transferred to PVDF membrane and probed with antibodies. Signals were detected by using ECL plus detection kit (Millipore; Catalogue no. WBKLS0500).

##### MTT assay

Cell viability was quantified by its ability to reduce tetrazolium salt 3-(4,5-dimethylthiazole-2ϒ)-2,5-diphenyl tetrasodium bromide (MTT) to colored formazan products. MTT reagent (5 mg/ml in PBS) was added to the cells at 1/10th volume of the medium to stain only the viable cells and incubated at 37 °C for 4 h. MTT solubilization buffer (0.01 M HCl, 10% SDS) of twofold volume was added to the cells, followed by incubation in the dark at 37 °C for 24 h. The absorbance was measured at 570 nm with Spectrostar Nano-Biotek, Lab Tech plate reader. Cell viability was expressed as the percentage of absorbance obtained in the control cultures.

##### Colony formation assay

The cells (*n* = 1000) were plated in triplicate in 60-mm tissue culture plates, and they were allowed to grow as a monolayer for 14 days. Cells were incubated in complete culture medium, with media changes after every 2–3 days. After 14 days, the cells were fixed with 4% paraformaldehyde for 1 h. The colonies were stained with 0.5% crystal violet (0.5 in 70% ethanol) for 1 h at room temperature, rinsed and air-dried. Surviving colonies with more than 50 cells were counted, and images were captured using a high-resolution Nikon D70 camera (Nikon, Tokyo, Japan). For quantification of the size of the colonies, ImageJ was used.

##### Wound healing assay

Cells were seeded at a high density, serum-starved for 16 h and wounded when the cells formed a confluent monolayer. Recovery of the wounds was recorded by using an inverted microscope equipped with CO_2_ and temperature maintenance accessory for 20 h with images captured at 10-min interval.

##### MNase digestion assay

Nuclei containing 2 mM CaCl_2_ were incubated for 2, 4, 6, 8 and 10 min with 5U MNase/mg of DNA at 37 °C in MNase digestion buffer (15 mM Tris–Cl pH 7.4, 15 mM NaCl, 2 mM CaCl_2_, 60 mM KCl, 15 mM β-ME, 0.5 mM spermidine, 0.15 mM spermine, 0.2 mM PMSF, protease and phosphatase inhibitors). The digestion was stopped by adding equal volume of 2 × lysis buffer (0.6 M NaCl, 20 mM EDTA, 20 mM Tris–Cl pH 7.5, 1% SDS). MNase-digested samples were treated with RNaseA (100 μg/ml) for 30 min at 37 °C followed by proteinase K (80 μg/ml) treatment for 2 h at 50 °C. The samples were extracted sequentially with phenol, phenol/chloroform and chloroform followed by ethanol precipitation at −20 °C. The precipitated DNA was recovered by centrifugation at 10,000*g* for 20 min. The DNA pellet was washed, air-dried and dissolved in TE buffer, and its concentration was determined by A260/A280 absorbance. MNase-digested samples were resolved on 1.8% 1XTAE agarose gel electrophoresis with 0.5 μg/ml ethidium bromide.

## Additional file

**Additional file 1: Figure S1.** Multiple alignment of all the H2A protein sequences in rat. **Figure S2**. RP-HPLC chromatogram of histones isolated from the rat liver tissue. **Figure S3.** Unique peptides identified for the H2A isoforms. **Figure S4**. Real-time PCR of H2A isoforms in the normal vs tumor liver tissues. **Figure S5**. H2A.1 and H2A.2 isoforms in the CL44 and CL38 cells. **Figure S6**. Migration and cell proliferation upon overexpression of the histone H2A isoforms. **Figure S7**. Sequence comparison of H2A isoforms. **Figure S8**. Approach for equilibrium unfolding analysis of histone dimers. **Figure S9**. Guanidine chloride induced denaturation of H2A-H2B dimer. **Figure S10**. RMSD of MDS. **Figure S11**. Primers.
