# Correction to: Histone isoform H2A1H promotes attainment of distinct physiological states by altering chromatin dynamics

**DOI:** 10.1186/s13072-018-0238-5

**Published:** 2018-11-16

**Authors:** Saikat Bhattacharya, Divya Reddy, Vinod Jani, Nikhil Gadewal, Sanket Shah, Raja Reddy, Kakoli Bose, Uddhavesh Sonavane, Rajendra Joshi, Duane Smoot, Hassan Ashktorab, Sanjay Gupta

**Affiliations:** 1 Epigenetics and Chromatin Biology Group, Gupta Lab, Cancer Research Institute, Advanced Centre for Treatment, Research and Education in Cancer (ACTREC), Tata Memorial Centre, Kharghar, Navi Mumbai, MH 410210 India; 2 Integrated Biophysics and Structural Biology Lab, Cancer Research Institute, Advanced Centre for Treatment, Research and Education in Cancer (ACTREC), Tata Memorial Centre, Kharghar, Navi Mumbai, MH 410210 India; 3 BTIS, Cancer Research Institute, Advanced Centre for Treatment, Research and Education in Cancer (ACTREC), Tata Memorial Centre, Kharghar, Navi Mumbai, MH 410210 India; 4 Homi Bhabha National Institute, Training School Complex, Anushakti Nagar, Mumbai, MH 400085 India; 5 Bioinformatics Group, Centre for Development of Advanced Computing (C‑DAC), University of Pune Campus, Pune, MH 411007 India; 6 Stowers Institute for Medical Research, Kansas City, MO 64110 USA; 7 Meharry Medical College, 1005 Dr DB Todd Jr Blvd, Nashville, TN 37208 USA; 8 Department of Medicine, Howard University Cancer Center, 2041 Georgia Avenue, NW, Suite 220, NW, Washington, DC 20059 USA

## Correction to: Epigenetics & Chromatin (2017) 10:48 10.1186/s13072-017-0155-z

After publication of this article [1], it was noticed Duane Smoot and Hassan Ashktorab who made and provided the cell line HFE145 were not included in the author list. RNA obtained from this cell line was used in one qRT-PCR experiment (Fig. 2d of the published manuscript).

The author list and author details have herewith been updated in this correction.

We apologize for the inconvenience caused.

## References

[1] Bhattacharya S, Reddy D, Jani V, Gadewal N, Shah S, Reddy R, Bose K, Sonavane U, Joshi R, Gupta S (2017). Histone isoform H2A1H promotes attainment of distinct physiological states by altering chromatin dynamics. Epigenet Chromatin.

