# Supplementary material for: Histone isoform H2A1H promotes attainment of distinct physiological states by altering chromatin dynamics
Source: Epigenetics Chromatin. 2017 Oct 18;10:48. doi: 10.1186/s13072-017-0155-z (PMC5648446; doi:10.1186/s13072-017-0155-z)
Supplement: Supplementary file 1 — Additional file 1: Figure S1. Multiple alignment of all the H2A protein sequences in rat. Figure S2. RP-HPLC chromatogram of histones isolated from the rat liver tissue. Figure S3. Unique peptides identified for the H2A isoforms. Figure S4. Real-time PCR of H2A isoforms in the normal vs tumor liver tissues. Figure S5. H2A.1 and H2A.2 isoforms in the CL44 and CL38 cells. Figure S6. Migration and cell proliferation upon overexpression of the histone H2A isoforms. Figure S7. Sequence comparison of H2A isoforms. Figure S8. Approach for equilibrium unfolding analysis of histone dimers. Figure S9. Guanidine chloride induced denaturation of H2A-H2B dimer. Figure S10. RMSD of MDS. Figure S11. Primers. [file 13072_2017_155_MOESM1_ESM.pdf]

Supplementary Information

```
H2A4          MSGRAKQGGKARAKAKSRSFRAGLQFPVGRVHRLLRQGNYAERIGAGTPVYLAAVLEYLT
H2A2B         MSGRGKQGGKARAKAKSRSSRAGLQFPVGRVHRLLRKGNYAERVGAGAPVYMAAVLEYLT
H2A2C         MSGRGKQGGKARAKAKSRSSRAGLQFPVGRVHRLLRKGNYAERVGAGAPVYMAAVLEYLT
H2A2A3        MSGRGKQGGKARAKAKSRSSRAGLQFPVGRVHRLLRKGNYAERVGAGAPVYMAAVLEYLT
H2A1F         MSGRGKQGGKARAKAKTRSSRAGLQFPVGRVHRLLYKGNYSERVGASAPVYLAAVLEYLT
H2A3          MSGRGKQGGKARAKAKSRSSRAGLQFPVGRVHRLLRKGNYSERVGAGAPVYLAAVLEYLT
H2A1K         MTGRGKQGGKARAKAKTRSSRAGLQFPVGRVHRLLRKGNYSERVGAGAPVYLAAVLEYLT
H2A1C         MSGRGKQGGKARAKAKTRSSRAGLQFPVGRVHRLLRKGNYSERVGAGAPVYLAAVLEYLT
H2AE-like     MSGRGKQGGKARAKAKTRSSRAGLQFPVGRVHRLLRKGNYSERVGAGAPVYLAAVLEYLT
H2A1IVx2      MSGRGKQGGKARAKAKTRSSRAGLQFPVGRVHRLLRKGNYSERVGAGAPVYLAAVLEYLT
H2A1IVx1      MSGRGKQGGKARAKAKTRSSRAGLQFPVGRVHRLLRKGNYSERVGAGAPVYLAAVLEYLT
H2A1N         MSGRGKQGGKARAKAKTRSSRAGLQFPVGRVHRLLRKGNYSERVGAGAPVYLAAVLEYLT
H2A1H         MSGRGKQGGKARAKAKTRSSRAGLQFPVGRVHRLLRKGNYAERVGAGAPVYLAAVLEYLT
              *:**.***********:** *************** :***:**:**.:***:********

H2A4          AEILELAGNAARDNKKTRIIPRHLQLAIRNDEELNKLLGRVTIAQGGVLPNIQAVLLPKK
H2A2B         AEILELAGNAARDNKKTRIIPRHLQLAVRNDEELNKLLGGVTIAQGGVLPNIQAVLLPKK
H2A2C         AEILELAGNAARDNKKTRIIPRHLQLAIRNDEELNKLLGKVTIAQGGVLPNIQAVLLPKK
H2A2A3        AEILELAGNAARDNKKTRIIPRHLQLAIRNDEELNKLLGKVTIAQGGVLPNIQAVLLPKK
H2A1F         AEILELAGNAARDNKKTRIIPRHLQLAIRNDEELNKLLGRVTIAQGGVLPNIQAVLLPKK
H2A3          AEILELAGNAARDNKKTRIIPRHLQLAIRNDEELNKLLGRVTIAQGGVLPNIQAVLLPKK
H2A1K         AEILELAGNAARDNKKTRIIPRHLQLAIRNDEELNKLLGRVTIAQGGVLPNIQAVLLPKK
H2A1C         AEILELAGNAARDNKKTRIIPRHLQLAIRNDEELNKLLGRVTIAQGGVLPNIQAVLLPKK
H2AE-like     AEILELAGNAARDNKKTRIIPRHLQLAIRNDEELNKLLGRVTIAQGGVLPNIQAVLLPKK
H2A1IVx2      AEILELAGNAARDNKKTRIIPRHLQLAIRNDEELNKLLGRVTIAQGGVLPNIQAVLLPKK
H2A1IVx1      AEILELAGNAARDNKKTRIIPRHLQLAIRNDEELNKLLGRVTIAQGGVLPNIQAVLLPKK
H2A1N         AEILELAGNAARDNKKTRIIPRHLQLAIRNDEELNKLLGRVTIAQGGVLPNIQAVLLPKK
H2A1H         AEILELAGNAARDNKKTRIIPRHLQLAIRNDEELNKLLGRVTIAQGGVLPNIQAVLLPKK
              ***************************:*********** ********************

H2A4          TESHHKSQTK
H2A2B         TDSHKPGKNK
H2A2C         TESHKAKSK-
H2A2A3        TESHHKAKGK
H2A1F         TESHHKSKGK
H2A3          TESHHKAKGK
H2A1K         TESHHKAKGK
H2A1C         TESHHKAKGK
H2AE-like     TESHHKAKGK
H2A1IVx2      TESHHKAKGK
H2A1IVx1      TESHHKAKGK
H2A1N         TESHHKAKGK
H2A1H         TESHHKAKGK
              *:**:  .
```

H2A.2 Isoforms (H2A2B, H2A2C, H2A2A3)

Identical proteins (H2A1C, H2AE-like, H2A1IVx2, H2A1IVx1, H2A1N)

Figure S1: Multiple alignment of all the H2A protein sequences in rat. Those names in bold depict that of H2A.2 Isoforms, highlighting M51 position. Remaining constitute H2A.1 species.

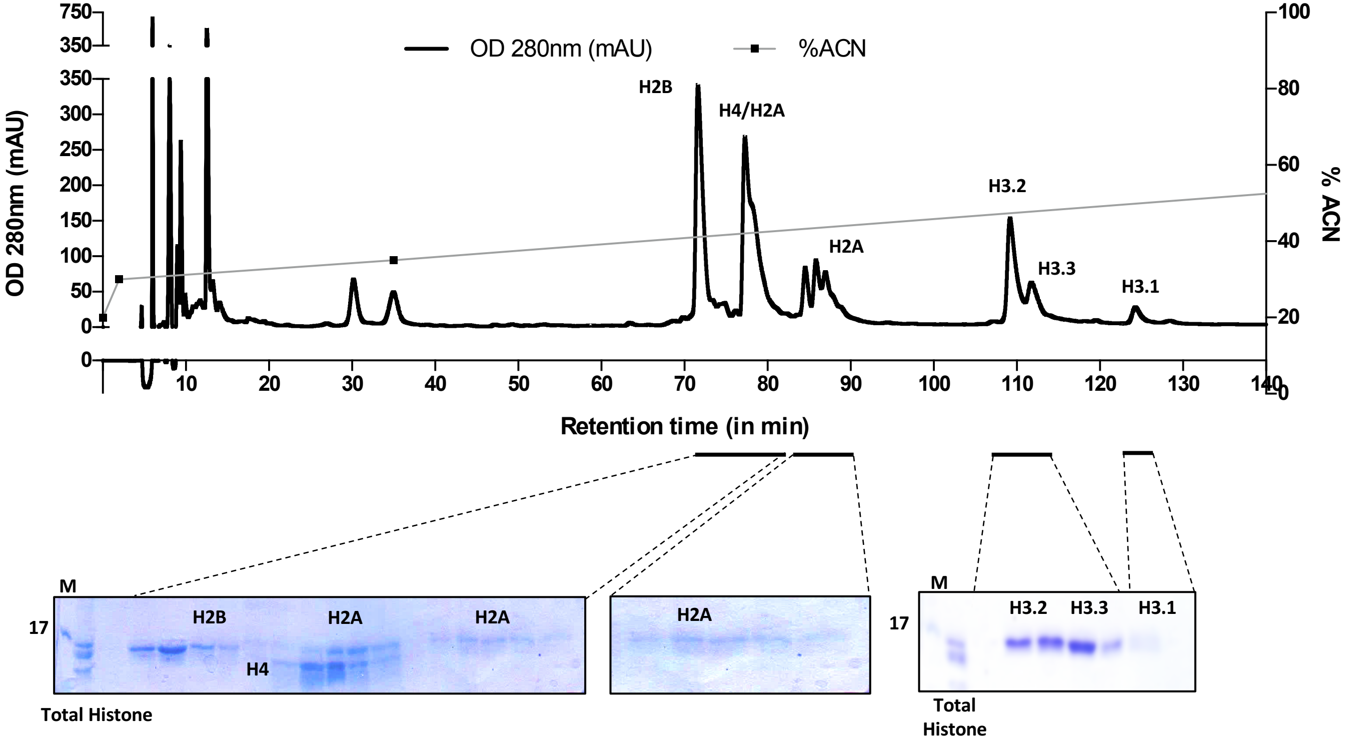

Figure S2: RP-HPLC chromatogram of histones isolated from the rat liver tissue. The different fractions eluted from the column were resolved on an 18% SDS-PAGE and stained with coomassie brilliant blue.

Isoform (Gene)	Theoretical MW	Peptides identified by MS
H2A1H (HIST1H2AH)	13988.30	ARAKAK**T**RSSRAGLQFPVGR<br>VGAGAPVY**L**AAVLEYLTAEILELAGNAAR<br>HLQLAIRNDEELNKLLG**R**
H2A2A3 (HIST2H2AA3)	13964.30	ARAKAK**S**RSSRAGLQFPVGR<br>VGAGAPVY**M**AAVLEYLTAEILELAGNAAR<br>HLQLAIRNDEELNKLLG**K**
H2A2C (HIST2H2AC)	13857.18	ARAKAK**S**RSSRAGLQFPVGR<br>VGAGAPVY**M**AAVLEYLTAEILELAGNAAR<br>HLQLAIRNDEELNKLLG**K**<br>KTESHKAK**S**K
H2A3 (HIST3H2A)	13990.28	ARAKAK**S**RSSRAGLQFPVGR<br>VGAGAPVY**L**AAVLEYLTAEILELAGNAAR<br>HLQLAIRNDEELNKLLG**R**
H2A1C (HIST1H2AC)	14004.30	ARAKAK**T**RSSRAGLQFPVGR<br>GNY**S**ERVGAGAPVYLAAVLEYLTAEILELAGNA<br>HLQLAIRNDEELNKLLG**R**
H2A1K (HIST1H2AK)	14018.33	ARAKAK**T**RSSRAGLQFPVGR<br>VGAGAPVY**L**AAVLEYLTAEILELAGNAAR<br>HLQLAIRNDEELNKLLG**R**
H2A1F (HIST1H2AF)	14057.32	ARAKAK**T**RSSRAGLQFPVGR<br>GNY**S**ERVGA**S**APVY**L**AAVLEYLTAEILELAGNA<br>HLQLAIRNDEELNKLLG**R**<br>KTESHK**S**KGK
H2A4 (HIST1H2AA)	14152.42	ARAKAK**S**RS**F**RAGLQFPVGR<br>**I**GA**GT**PVY**L**AAVLEYLTAEILELAGNAAR<br>HLQLAIRNDEELNKLLG**R**<br>KTESHHK**SQT**K
H2A2B (HIST2H2AB)	13868.12	ARAKAK**S**RSSRAGLQFPVGR<br>VGAGAPVY**M**AAVLEYLTAEILELAGNAAR<br>HLQLAIRNDEELNKLLG**G**VTIAQGGVLP

Figure S3: Unique peptides identified for the H2A isoforms. The unique residues are highlighted in bold.

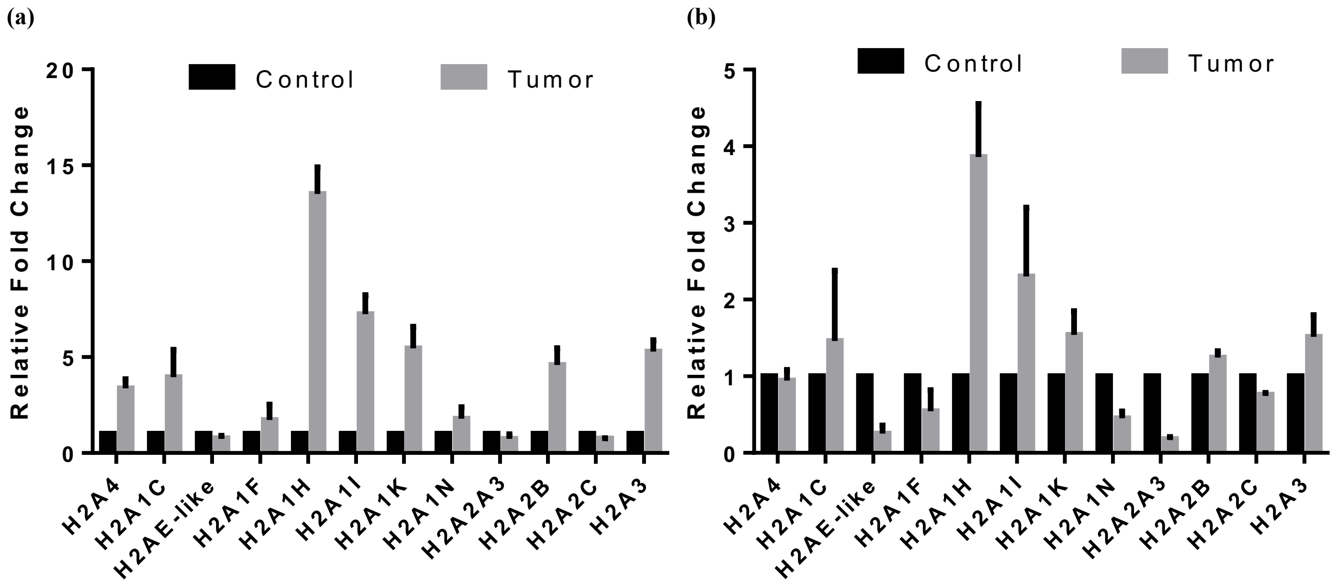

Figure S4: Real-time PCR of H2A.1 and H2A.2 isoforms in the normal vs tumor liver tissues. (a) Normalized to GAPDH. (b) Normalized to H4. The error bars represent SEM of three independent experiments.

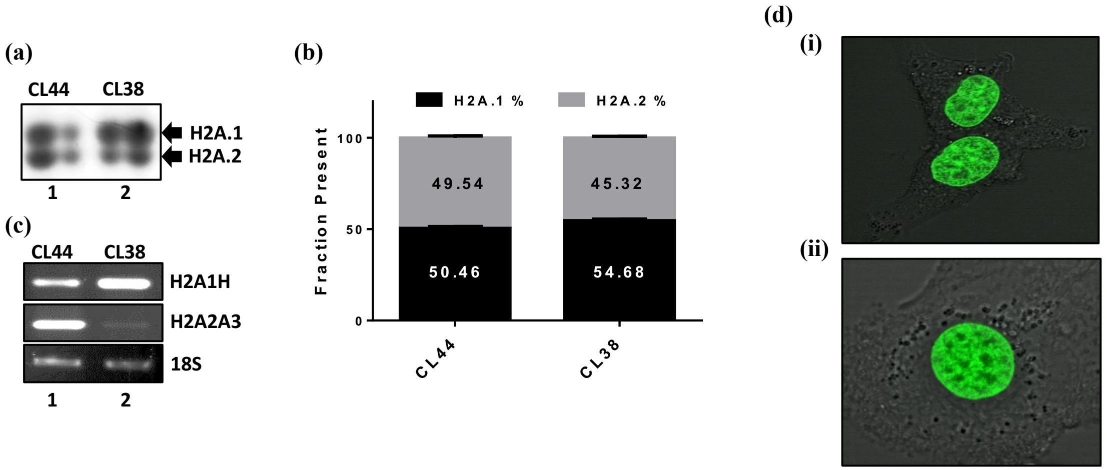

Figure S5: H2A.1 and H2A.2 isoforms in the CL44 and CL38 cells. (a) AUT-PAGE gel showing the basal levels of H2A.1 and H2A.2 (arrows marked) in the CL44 and CL38 cells. (b) Quantitative data of the AUT-PAGE showing the relative levels of H2A.1/H2A.2. Error bars (3 independent experiments) are of SEM. (c) Agarose gel showing the expression of H2A1H and H2A2A3 isoforms in the cell lines by semi-quantitative RT-PCR. (d) Localisation/distribution of (i) YFP-H2A1H (ii) YFP-H2A2A3 in nucleus of the CL38 cells.

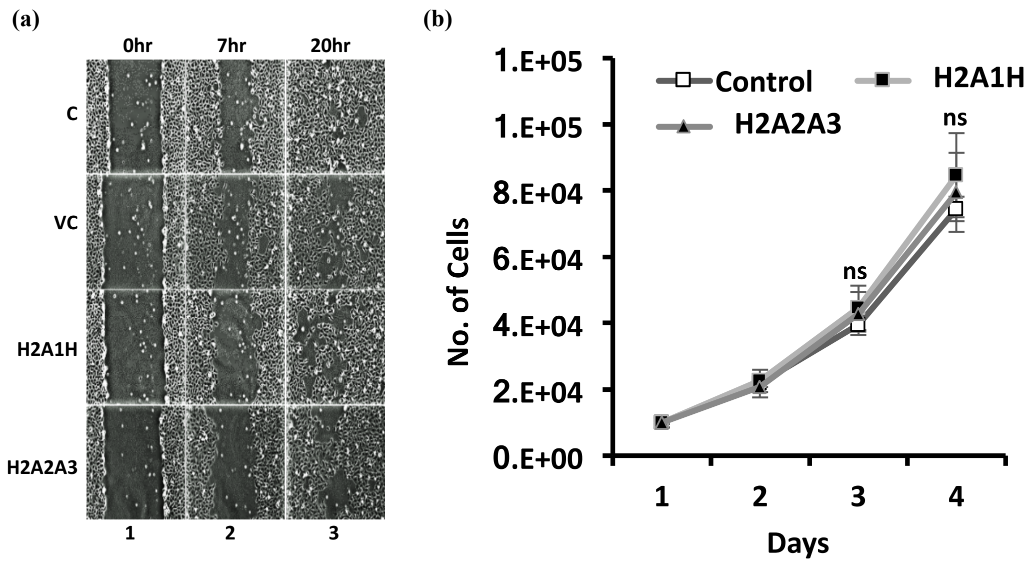

Figure S6: Migration and cell proliferation upon overexpression of the histone H2A isoforms, H2A1H and H2A2A3. (a) Wound healing assay for analysis of the changes in migration potential of the CL38 cells upon overexpression of the H2A isoforms. C- Control (untransfected), VC- Vector Control. (b) Cell proliferation monitored by MTT assay upon overexpression of H2A isoforms in CL44 cells. Error bar represents SEM of 6 independent experiments.

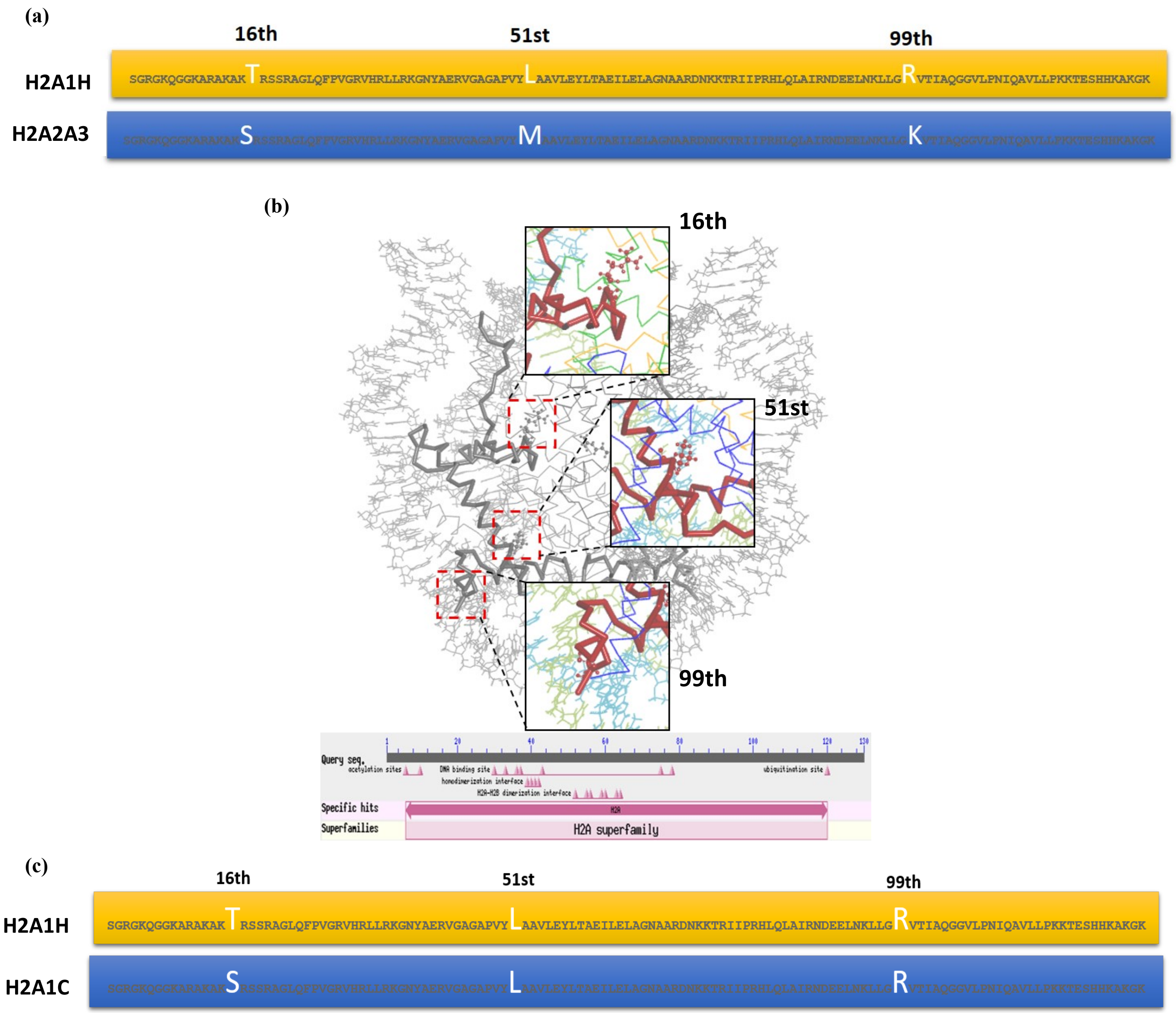

Figure S7: (a) Alignment of the H2A1H and H2A2A3 protein sequences highlighting the differential residues. (b) Nucleosome model depicting the location of the three differential amino acids between H2A1H and H2A2A3. (c) Alignment of H2A1H of rat with H2A1C of humans.

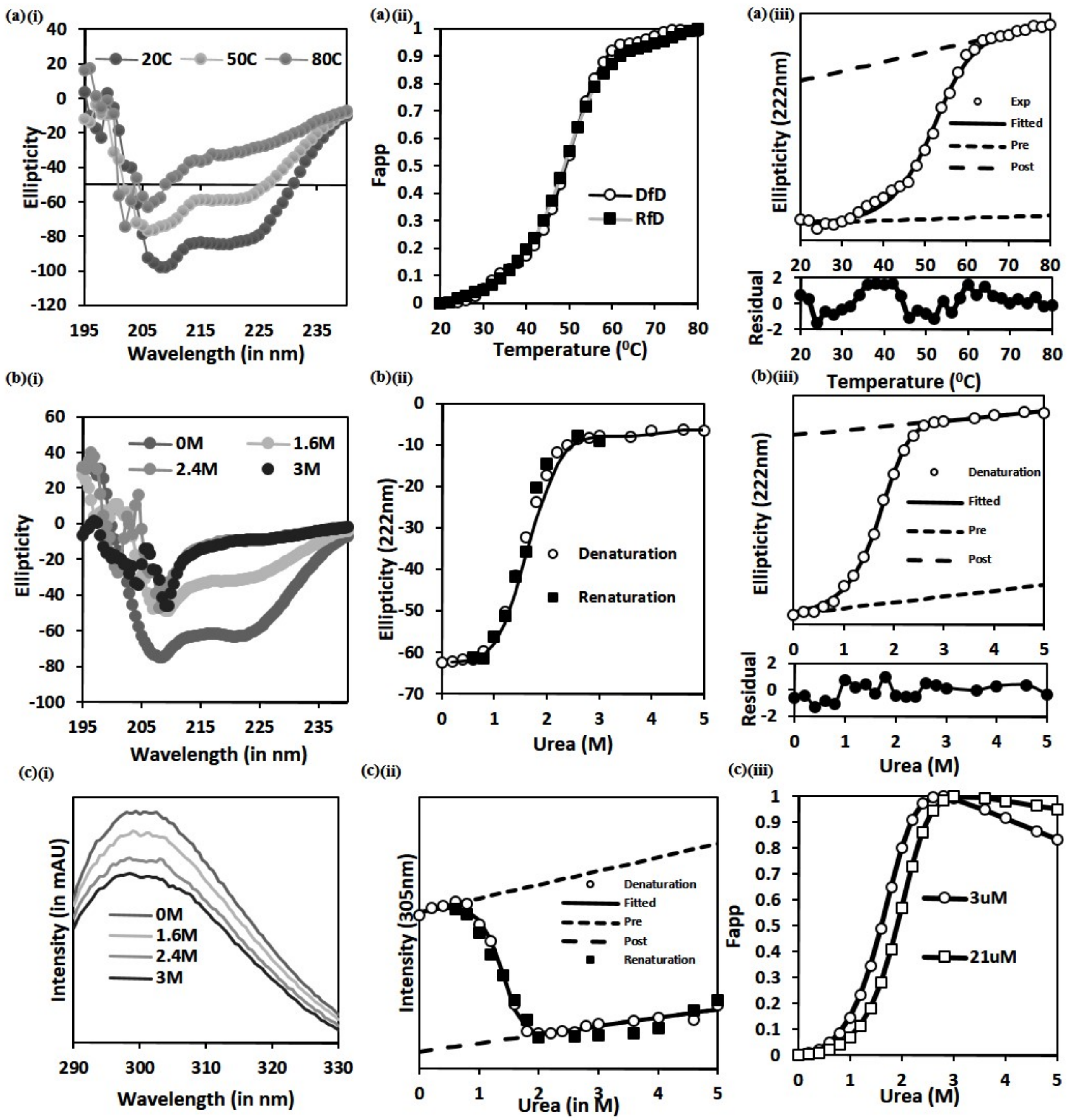

Figure S8: Approach for equilibrium unfolding analysis for histone dimers. (a)(i) Thermal denaturation followed by CD spectra. (ii) (iii) Graph showing the kinetics of denaturation and renaturation. (b) Urea denaturation followed by CD spectra. (ii) (iii) Graph showing the kinetics of denaturation and renaturation. (c)(i) Urea denaturation followed by fluorescence spectroscopy. (ii) Graph showing the kinetics of denaturation and renaturation. (iii) Spectra taken at different concentration of proteins.

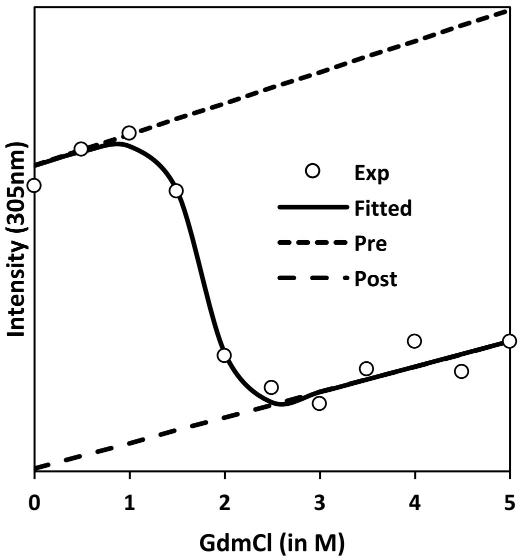

Figure S9: Guanidine chloride induced denaturation followed by CD spectra of histone dimer.

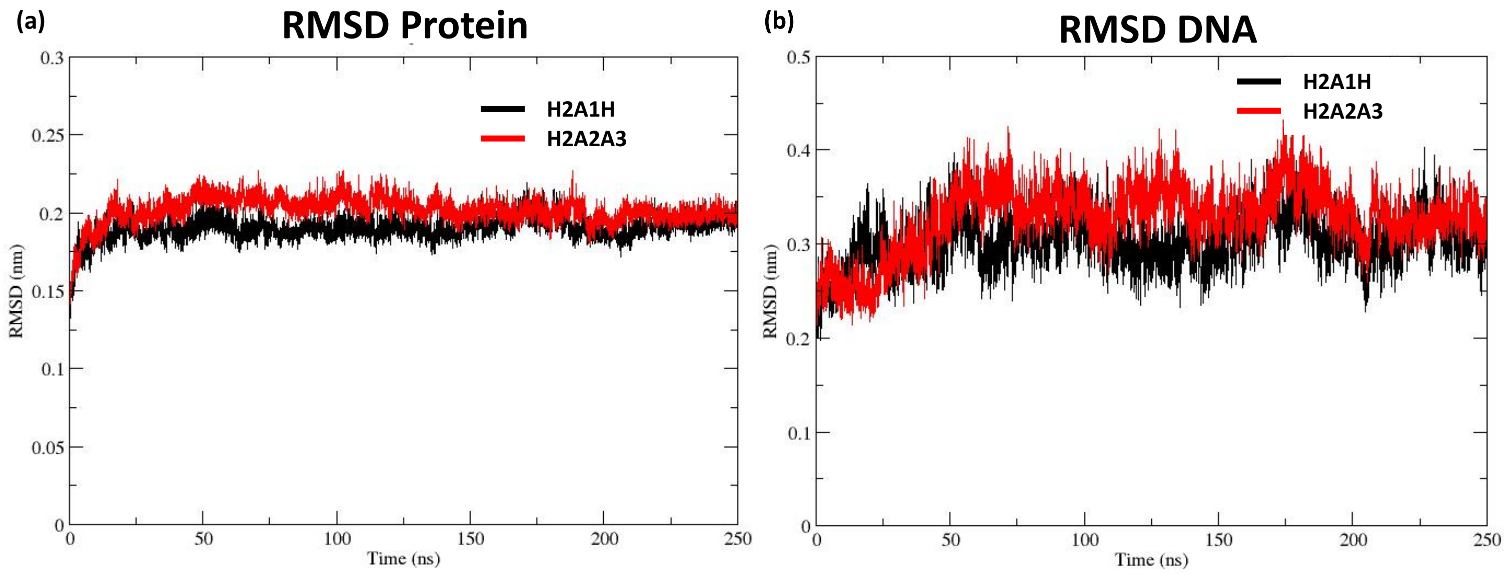

Figure S10: RMSD of (a) protein and (b) DNA throughout the 250 ns time frame of MDS.

Gene	Protein	Species	Primer Orientation	Primer Sequence (5' - 3')
HIST1H2AC	H2A1C	Homo sapiens	Forward	GGTGATTTTTGTCTGATTG
			Reverse	CTGCGTAGTTGCCTTTA
HIST2H2AA3	H2A2A3	Homo sapiens	Forward	CGGTGACTACTATCGCTGT
			Reverse	AGCCATGTAGACGGGC
HIST1H2AH	H2A1H	Rattus norvegicus	Forward	CTGTGCTGGAGTACCTGACG
			Reverse	TGTGGTGGCTCTCAGTCTTC
HIST2H2AA3	H2A2A3	Rattus norvegicus	Forward	GAAGACGGAGAGCCACCATA
			Reverse	GGAAGAGTAGGGCACACGAC
Ki67	Ki67	Rattus norvegicus	Forward	AGACGTGACTGGTTCCCAAC
			Reverse	ACTGCTTCCCGAGAACTGAA
PCNA	PCNA	Rattus norvegicus	Forward	TCACAAAAGCCACTCCACTG
			Reverse	CATCTCAGAAGCGATCGTCA
18S	18S	Rattus norvegicus	Forward	CGCGGTTCTATTTTGTTGGT
			Reverse	AGTCGGCATCGTTTATGGTC
H4	H4	Rattus norvegicus	Forward	ATGTCTGGCAGAGGAAAGGGTG
			Reverse	CTAGCCTCCGAAGCCGTACA
GAPDH	GAPDH	Rattus norvegicus	Forward	GGATTTGGTCGTATTGGGCG
			Reverse	ATCGCCCCACTTGATTTTGG
HIST1H2AC	H2A1C	Rattus norvegicus	Forward	CACCACAAGGCCAAGGGAAA
			Reverse	GACTTGCGGTGCCATCTAGG
HIST1H2AE	H2AE-like (H2A1C)	Rattus norvegicus	Forward	AGCTCCTCACACTTCAGCATAA
			Reverse	GCTCCGAGTAGTTGCCCTTG
HIST1H2AA	H2A4	Rattus norvegicus	Forward	AGAGCAGGTTTGCAGTTTCCT
			Reverse	TGTTATCCCTCGCCGCATTG
HIST2H2AB	H2A2B	Rattus norvegicus	Forward	CTAGGAGGAGTCACCATTGCC
			Reverse	TTTCCAGGCTTATGACTATCCGT
HIST1H2AF	H2A1F	Rattus norvegicus	Forward	GTGCACCGTCTGCTTTACAA
			Reverse	TGGCTCTCGGTCTTTTTGGG
HIST1H2AI	H2A1I (H2A1C)	Rattus norvegicus	Forward	GGCGTTCTGCCAAACATCC
			Reverse	AAGAGCCTTTGGTGATCCCTG
HIST1H2AN	H2A1N (H2A1C)	Rattus norvegicus	Forward	ACCTTACCTTTTCCACTTCCATCT
			Reverse	ATGCAGAAGCGTAGAGCCAA
HIST2H2AC	H2A2C	Rattus norvegicus	Forward	ACCAACCTAGCTTCATCAGAGT
			Reverse	CCGCGGCCAGACATAACTAA
HIST3H2A	H2A3	Rattus norvegicus	Forward	AACGACGAGGAGCTCAACAA
			Reverse	GTAACTGGCGACAGGCTCAG
HIST1H2AK	H2A1K	Rattus norvegicus	Forward	ACGAGGAGCTCAACAAGCTG
			Reverse	TGGTGGCTCTCGGTCTTCTT
PanH4	H4	Rattus norvegicus	Forward	GCATCTCCGGCCTCATCTAC
			Reverse	ACATCCATGGCAGTGACAGT

Figure S11: List of primers.
